# Immunization of Mice with Virus-Like Vesicles of Kaposi Sarcoma-Associated Herpesvirus Reveals a Role for Antibodies Targeting ORF4 in Activating Complement-Mediated Neutralization

**DOI:** 10.1128/jvi.01600-22

**Published:** 2023-02-09

**Authors:** Alex K. Lam, Romin Roshan, Wendell Miley, Nazzarena Labo, James Zhen, Andrew P. Kurland, Celine Cheng, Haigen Huang, Pu-Lin Teng, Claire Harelson, Danyang Gong, Ying K. Tam, Caius G. Radu, Marta Epeldegui, Jeffrey R. Johnson, Z. Hong Zhou, Denise Whitby, Ting-Ting Wu

**Affiliations:** a Department of Molecular and Medical Pharmacology, David Geffen School of Medicine, University of California, Los Angeles, California, USA; b AIDS and Cancer Virus Program, Frederick National Laboratory for Cancer Research, Frederick, Maryland, USA; c Department of Microbiology, Immunology, and Molecular Genetics, David Geffen School of Medicine, University of California, Los Angeles, California, USA; d Department of Microbiology, Icahn School of Medicine at Mount Sinai, New York, New York, USA; e Acuitas Therapeutics, Vancouver, British Columbia, Canada; f Department of Obstetrics and Gynecology, David Geffen School of Medicine, University of California, Los Angeles, California, USA; Lerner Research Institute, Cleveland Clinic

**Keywords:** KSHV, vaccine, antibody function, complement, neutralizing antibodies

## Abstract

Infection by Kaposi sarcoma-associated herpesvirus (KSHV) can cause severe consequences, such as cancers and lymphoproliferative diseases. Whole inactivated viruses (WIV) with chemically destroyed genetic materials have been used as antigens in several licensed vaccines. During KSHV productive replication, virus-like vesicles (VLVs) that lack capsids and viral genomes are generated along with virions. Here, we investigated the immunogenicity of KSHV VLVs produced from a viral mutant that was defective in capsid formation and DNA packaging. Mice immunized with adjuvanted VLVs generated KSHV-specific T cell and antibody responses. Neutralization of KSHV infection by the VLV immune serum was low but was markedly enhanced in the presence of the complement system. Complement-enhanced neutralization and complement deposition on KSHV-infected cells was dependent on antibodies targeting viral open reading frame 4 (ORF4). However, limited complement-mediated enhancement was detected in the sera of a small cohort of KSHV-infected humans which contained few neutralizing antibodies. Therefore, vaccination that induces antibody effector functions can potentially improve infection-induced humoral immunity. Overall, our study highlights a potential benefit of engaging complement-mediated antibody functions in future KSHV vaccine development.

**IMPORTANCE** KSHV is a virus that can lead to cancer after infection. A vaccine that prevents KSHV infection or transmission would be helpful in preventing the development of these cancers. We investigated KSHV VLV as an immunogen for vaccination. We determined that antibodies targeting the viral protein ORF4 induced by VLV immunization could engage the complement system and neutralize viral infection. However, ORF4-specific antibodies were seldom detected in the sera of KSHV-infected humans. Moreover, these human sera did not potently trigger complement-mediated neutralization, indicating an improvement that immunization can confer. Our study suggests a new antibody-mediated mechanism to control KSHV infection and underscores the benefit of activating the complement system in a future KSHV vaccine.

## INTRODUCTION

Kaposi sarcoma-associated herpesvirus (KSHV) is the etiological agent for Kaposi sarcoma (KS), a malignancy that manifests as lesions that mainly consist of endothelial cells on the skin, lymph nodes, lungs, and digestive tract (1). While the occurrence of KS is low overall in the United States, at a rate of 4.5 cases per million people in 2017, it could be up to 500 times higher in transplant patients and in people living with human immunodeficiency virus (2, 3). KSHV is prevalent in regions of endemicity such as sub-Saharan Africa, where over 50% of individuals are infected (1). KSHV also causes lymphoproliferative disorders, including primary effusion lymphoma, multicentric Castleman disease, and KSHV inflammatory cytokine syndrome. The clear link between KSHV infection and KSHV-associated cancers demonstrates a distinct benefit from a prophylactic vaccine: stopping KSHV infection to eliminate KSHV-associated disorders. This would be most beneficial in resource-limited sub-Saharan Africa, where KSHV is endemic.

In general, the correlate of protection of prophylactic viral vaccines is the generation of neutralizing antibodies, which bind the viral attachment and entry proteins to prevent infection (4). Antibodies also have effector functions that aid in antiviral immunity. One such effector function is the engagement of the classical complement system, which activates an enzymatic cascade after binding to an antibody complex on the surface of a pathogen or infected cell. This can lead to the development of the membrane attack complex (MAC) that forms pores in the membrane to neutralize or kill the pathogen or infected cell (5). The importance of effector functions has also been suggested by studies of mother-child transmission pairs for KSHV and Epstein-Barr virus (EBV). KSHV-seropositive mothers of children who did not seroconvert had higher average serum antibodies than mothers of seroconverted children. However, there was no difference in neutralizing antibody levels between these two groups of mothers, implicating a role of effector functions (6). Moreover, a study of EBV acquisition in infants found no evidence for protection from neutralizing maternal antibodies against EBV infection, indicating a role of nonneutralizing antibody functions in protection (7).

Several licensed vaccines are based on whole inactivated viruses (WIV) with the goal of inducing antibodies targeting surface proteins to prevent infection. To allow for presentation of an entire repertoire of surface proteins, another vaccine approach has been developed based on mutant viruses that make only noninfectious particles lacking viral genomes. For EBV, these noninfectious virus-like particles (VLPs) are generated from EBV mutants deficient in viral genome packaging or viral maturation (8, 9). Mice immunized with EBV VLPs produce virus-specific antibody and T cell responses (8). Notably, wild-type herpesviruses can produce noninfectious particles devoid of capsids and viral DNA in addition to virions (10). We previously showed that KSHV also produces analogous noninfectious particles, and we referred them to as virus-like vesicles (VLVs) (11). KSHV VLVs can be produced from a cell line stably infected with a capsid-deficient mutant that does not form virions (11). KSHV VLVs contain the same set of envelope proteins as virions but lack capsid and capsid-associated proteins. Moreover, these VLVs do not have encapsidated viral genomes, largely eliminating the oncogenic risk of latent infection. We hypothesized that VLVs hold the potential to present a broad spectrum of structural proteins to the immune system.

Generally, immunization with protein preparations faces the challenge of poor immunogenicity, and these formulations require adjuvants to obtain substantial immune responses (12). Adjuvants can promote delivery of vaccine antigens and/or modulate the immune system to better trigger adaptive responses. For example, traditional adjuvants such as aluminum salts and oil-in-water emulsions induce “danger” signals and recruit innate immune cells to improve antigen presentation. Newer adjuvants trigger innate immune responses by signaling through toll-like receptors (TLRs), such as monophosphoryl lipid A binding to TLR4 or single-stranded RNA binding to TLR7 and TLR8. Recently, lipid nanoparticles (LNP) have emerged as a contributor of adjuvant activity to mRNA-based vaccines, potentially through interleukin 1 beta (IL-1β) and IL-6 signaling (13, 14). Furthermore, both empty LNP and LNP encapsulating a TLR ligand have been shown to be potent adjuvants for protein-based vaccines (15, 16). Thus, the multitude of adjuvants that have been developed provides a wide base for improving responses to protein-based KSHV vaccines.

In this study, we investigated the immunogenicity of KSHV VLVs produced from a KSHV mutant deficient in capsid formation. We show that KSHV VLVs coinjected with an LNP-based adjuvant into mice elicit virus-specific T cells alongside antibody responses with low neutralizing activity. The targets of VLV-induced antibodies include viral open reading frame 4 (ORF4), a known complement control protein (17). These anti-ORF4 antibodies are primarily directed against the short consensus repeat (SCR) domain and mediate complement-enhanced neutralization of KSHV infection and complement deposition on KSHV-infected cells by the VLV immune sera. However, we did not detect robust complement-enhanced neutralization in the sera from a cohort of humans infected with KSHV, suggesting a potential benefit that immunization can confer over infection-generated immunity. Our results warrant future investigations into the mechanisms of complement-mediated control of KSHV infection and highlight a potential advantage of engaging the complement system in a future KSHV vaccine.

## RESULTS

### Production of VLVs with a KSHV mutant deficient in capsid formation.

**FIG 1:**
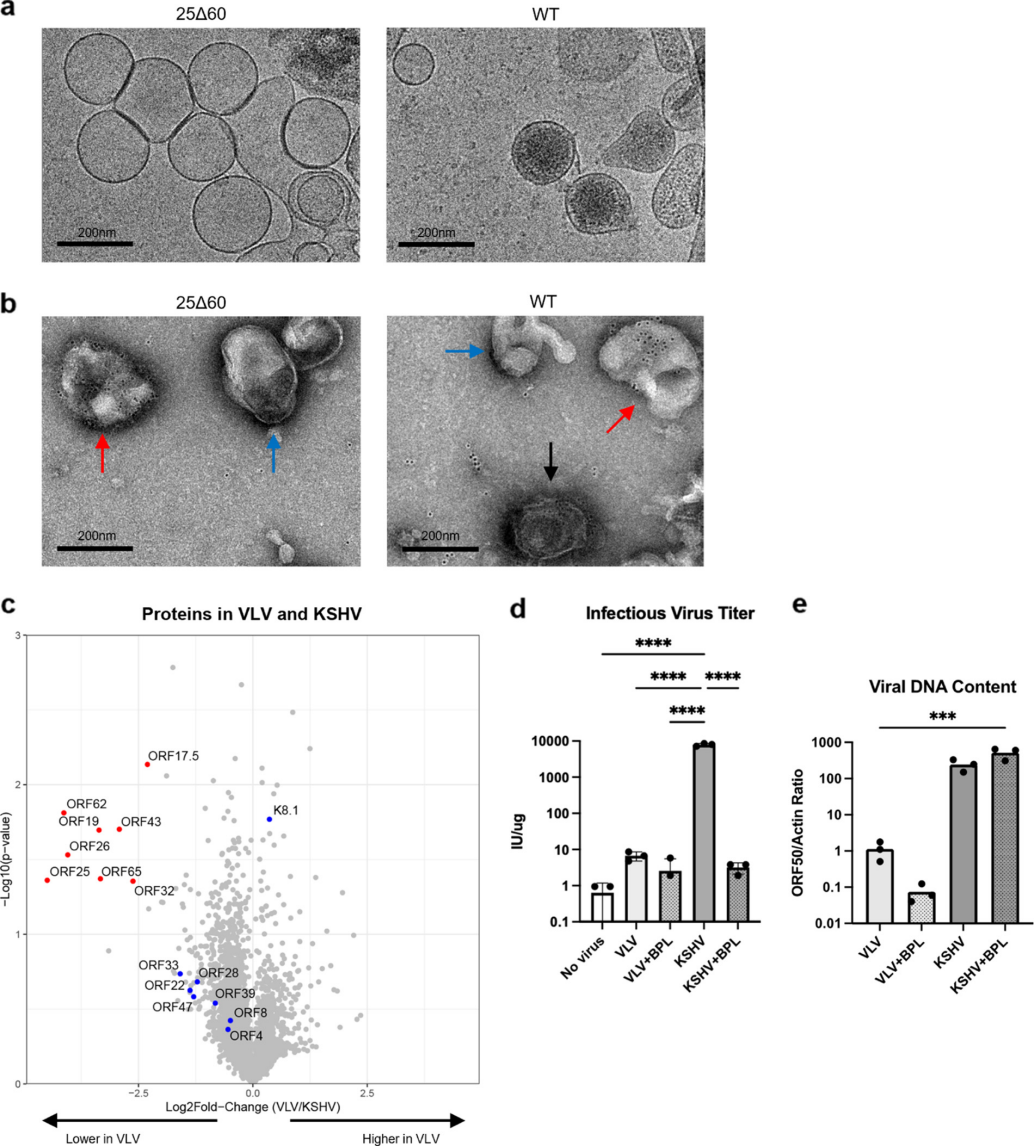
VLVs generated from a KSHV mutant defective in capsid formation presented KSHV antigens without viral DNA or infectivity. (a) Cryo-electron micrographs of VLVs and KSHV virions obtained from reactivated iSLK 25Δ60 or WT cells. (b) Negative-stain micrographs of VLVs and KSHV virions labeled with K8.1 antibodies and immunogold beads. Red arrows indicate labeled VLVs, blue arrows indicate unlabeled vesicles, and black arrows indicate labeled virions. Vesicles were considered positively labeled if they were over 100 nm in diameter and were labeled with more than five gold beads. (c) Volcano plot showing proteins found in VLV and KSHV virion preparations. Differentially present proteins (log_2_ fold change of less than −2 and *P* < 0.05) are highlighted in red, and other viral proteins of interest are highlighted in blue. Statistical analysis for MS data was performed as described in Materials and Methods, with 3 preparations of VLV or KSHV. (d) Infectious virus titers (means and standard deviations) from VLV and KSHV preparations or preparations inactivated with BPL. Statistical analysis used ordinary one-way analysis of variance (ANOVA) with Tukey’s test for multiple comparisons (*N* = 3 independent infection assays). ****, *P* < 0.0001. (e) Ratio of viral ORF50 DNA to actin in VLV and KSHV preparations from carrier DNA used during isolation (means and standard deviations). Statistical analysis used ordinary one-way ANOVA with Dunnett’s test for multiple comparisons for the VLV group (*N* = 3 DNA extractions). Only the comparison with KSHV+BPL was significant (***, *P* < 0.001).

To produce VLVs without virions, we utilized a previously described KSHV mutant deficient in capsid formation due to a 60-amino-acid deletion in the major capsid protein (ORF25Δ60) (18). SLK cells, a renal cell carcinoma cell line (19), were engineered to express doxycycline (DOX)-inducible RTA, the immediate-early protein that drives the lytic replication of KSHV (20). The resultant cell line, iSLK, was used to establish KSHV stably infected cells harboring the latent 25Δ60 viral genome. These cells were treated with sodium butyrate and DOX to reactivate the viral lytic cycle, resulting in the production of VLVs that could be isolated from the culture supernatant by ultracentrifugation. We characterized VLV samples and compared them to KSHV virion samples prepared in a similar manner using iSLK cells harboring wild-type (WT) latent KSHV (21). Under cryo-electron microscopy, we observed vesicles around 200 nm in diameter in samples from iSLK-25Δ60 (Fig. 1a), and these vesicles were referred to as VLVs. In the WT sample, we observed virions that contained capsids, indicated by dense cores, in addition to VLVs. To demonstrate the presence of KSHV antigens, we performed immunogold staining for viral glycoprotein K8.1. Both samples contained vesicles that were labeled with gold particles (Fig. 1b, red arrows), and labeled virions were detected in the WT sample (Fig. 1b, black arrow). We further characterized the protein contents using mass spectrometry (MS). As expected, the 25Δ60 sample contained comparable levels of viral envelope proteins, such as ORF8 (gB) and K8.1, as in the WT sample (Fig. 1c, blue dots) and much lower levels of capsid-associated proteins, such as ORF25 and ORF65 (Fig. 1c, red dots). We also identified other viral proteins, including tegument proteins and cellular proteins, that were previously reported in VLVs (see Tables S1 and S2 in the supplemental material) (11).

To demonstrate that VLVs generated from iSLK-25Δ60 were noninfectious, we incubated them with HEK293 cells. KSHV derived from BAC16 contains expression cassettes for green fluorescent protein (GFP) and hygromycin resistance in the genome (21). Thus, cells infected with BAC16-derived viruses express GFP and are resistant to hygromycin. Using flow cytometry, we detected a trace amount of GFP-positive events (<10 per 10,000 events) from cells incubated with VLV, KSHV virions inactivated with beta-propiolactone (BPL; “KSHV+BPL”), and BPL-treated VLV (VLV+BPL) (Fig. 1d). Moreover, this minor level of GFP-positive cells was not significantly different from that obtained from the no-virus sample, indicating a possible background signal of flow cytometry. On the other hand, when incubated with an equal protein amount of WT KSHV, ~8,000 GFP-positive cells were detected per microgram of protein. In addition, we also performed hygromycin selection. While cells continued to proliferate after incubation with WT KSHV, we found no surviving cells after incubation with VLVs prepared from the 25Δ60 virus, confirming the lack of infectivity (data not shown). Finally, we isolated DNA from 25Δ60-derived VLVs and WT KSHV virions to assess the levels of viral DNA. We found that VLVs had >100-fold less viral DNA than the KSHV virions, which was expected due to the predicted inability of the 25Δ60 virus to encapsidate the viral genome (Fig. 1e). Overall, we concluded that the 25Δ60-derived VLVs contained a repertoire of KSHV envelope proteins similar to KSHV virions but with a reduced risk of latent infection.

### VLV immunization generates virus-specific antibodies and T cells in mice.

Next, we examined the immunogenicity of VLVs and compared them to inactivated KSHV virions. To control the effect of BPL inactivation, we treated VLVs with BPL employing the same protocol used to inactivate KSHV virions. Mice were immunized intraperitoneally with 2 µg of BPL-treated VLV (VLV+BPL) or BPL-treated KSHV virions (KSHV+BPL) three times at an interval of 3 weeks. Immune serum collected at 14 days after the third immunization was assessed for KSHV-specific antibodies (Fig. S1a). As K8.1 is one of the most common targets for KSHV-specific antibodies in human serum (22), we ran an enzyme-linked immunosorbent assay (ELISA) using purified K8.1 protein. VLV immunization elicited a lower level of K8.1 antibodies than did inactivated KSHV virions (Fig. S1b). To identify other targets of antibody responses, we utilized a bead-based multiplexed assay that included 62 KSHV proteins, and we identified ORF4, in addition to K8.1, as a target of antibodies generated by VLV immunization (Fig. S1c). Immunization of KSHV+BPL generated a broader antibody response to multiple proteins besides K8.1 and ORF4, such as tegument protein ORF38 and the small capsid protein ORF65.

**FIG 2:**
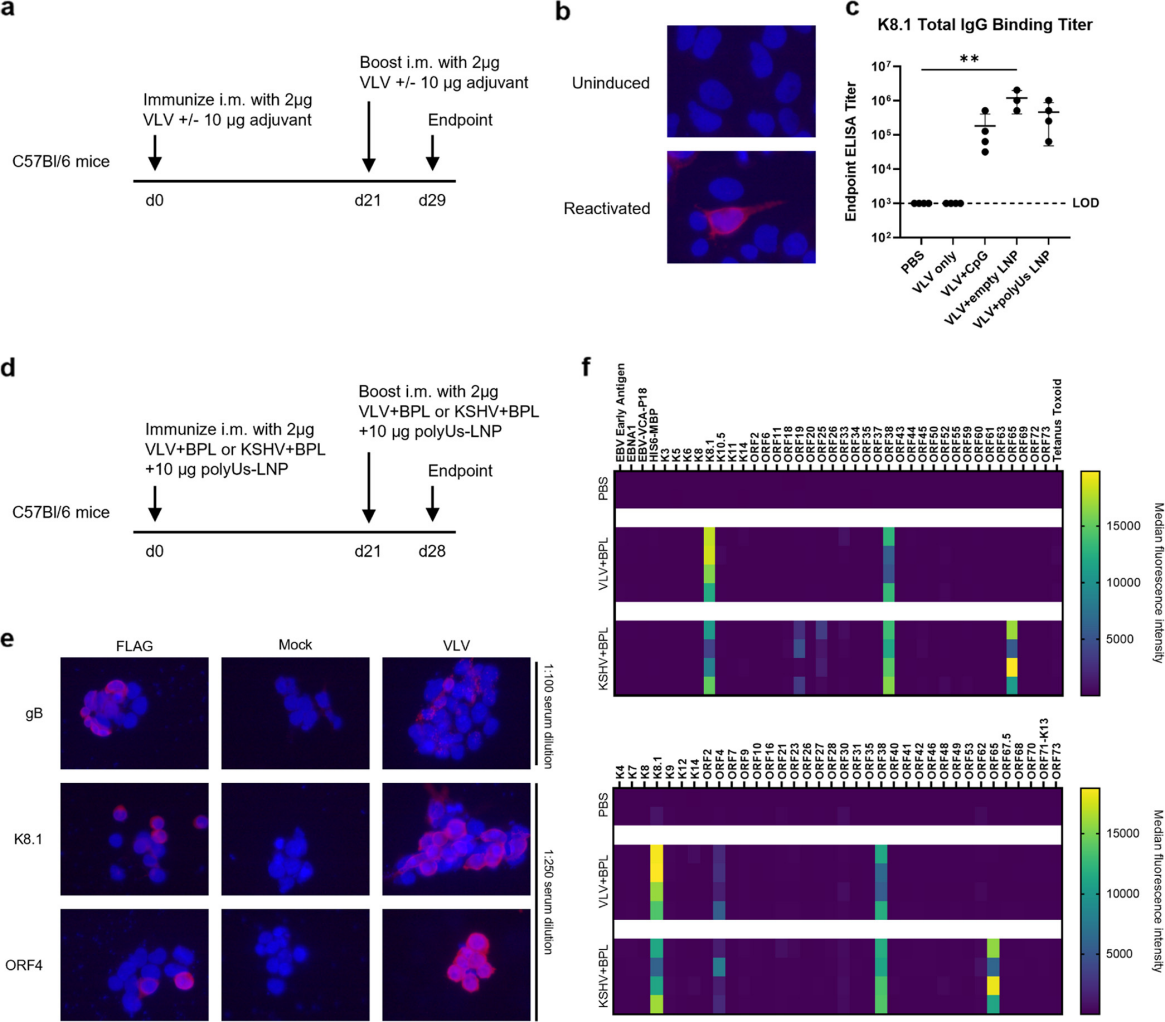
Mice immunized intramuscularly with adjuvanted VLVs generated virus-specific antibody responses. (a) Immunization scheme to study immunogenicity of VLV with adjuvants and the immune sera collected at the endpoint used for results shown in panels b and c. (b) Immunofluorescence images of uninduced or reactivated iSLK WT cells stained with the pooled VLV+poly(U)s-LNP immune serum at a 1:250 dilution. (c) Endpoint K8.1 ELISA titers of VLV immune sera. LOD, limit of detection. Statistical analysis used an ordinary one-way ANOVA with Dunnett’s test for multiple comparisons to the PBS mock-immunized group. **, *P* < 0.01. Means with standard deviations are shown (*N* = 3 to 4 per group). (d) Immunization scheme to compare VLV+BPL and KSHV+BPL adjuvanted with poly(U)s-LNP adjuvant and the immune sera collected at the endpoint used for results shown in panels e and f. (e) Immunofluorescence images of 293T cells expressing indicated KSHV glycoproteins stained with pooled mock or VLV+BPL immune serum at the indicated dilutions. (f) Heatmaps showing antigen binding of immune sera at a 1:200 dilution in a bead-based multiplex KSHV antibody assay. Each heatmap represents a different set of beads with some overlapping antigens. Each row represents a different mouse.

We also assessed the immune responses by immunizing mice via a clinically relevant route, intramuscular administration. Unlike inactivated KSHV virions, VLVs contain little viral DNA, a major class of pathogen-associated molecular patterns (PAMPs). Upon binding to PAMPs, pattern recognition receptors initiate downstream signaling to activate a variety of innate immune responses which play an essential role in instructing adaptive immunity. Therefore, to increase the immunogenicity of VLVs, we coadministered them with one of three types of adjuvants: (i) a CpG-containing oligodeoxynucleotide (CpG ODN), an agonist of toll-like receptor 9 (TLR9) (23); (ii) empty lipid nanoparticles (LNP); and (iii) LNP encapsulating phosphothioate-linked polyuridlyic acid [poly(U)s], an agonist of TLR7 (24). Mice were immunized twice with 2 µg of VLVs alone or in combination with 10 µg of one type of adjuvant at a 3-week interval (Fig. 2a). We then examined serum antibody responses at 8 days after the second immunization. By immunofluorescence assay (IFA), we confirmed that serum from immunized mice recognized reactivated KSHV-infected iSLK cells and not uninduced cells, suggesting that it contained antibodies that targeted antigens expressed on the cell surface during the lytic viral cycle (Fig. 2b). By K8.1 ELISA, we found that immunization of VLVs alone gave results similar to phosphate-buffered saline (PBS)-immunized mice, and all three types of adjuvants enhanced the immunogenicity of VLVs to elicit K8.1-specific antibody responses (Fig. 2c). In a separate intramuscular immunization experiment (Fig. 2d), we compared the immunogenicity of VLV+BPL with KSHV+BPL adjuvanted with poly(U)s-LNP. Using a small panel of expression plasmids for viral envelope proteins, we showed that the VLV+BPL immune sera contained antibodies that recognized surface expression of K8.1, ORF4, and gB (Fig. 2e), but not gH/gL, gM/gN, or ORF28 (Fig. S2a). VLVs elicited a similar level of K8.1 binding antibodies to inactivated virions (Fig. S2b). In addition, we also examined the antibody profiles using a bead-based multiplexed assay. Other than the two envelope proteins, K8.1 and ORF4, VLV+BPL adjuvanted with poly(U)s-LNP also induced significant antibodies against ORF38 (Fig. 2f), which were only weakly detected when VLVs were administered intraperitoneally without adjuvants (Fig. S1c). The antibody responses to these three proteins were comparable between immunization of VLVs and inactivated virions. As we expected, VLVs did not generate antibodies against the small capsid protein ORF65, while inactivated virions did (Fig. 2f). Thus, when codelivered with adjuvants via an intramuscular route, VLVs and inactivated virions share a similar capacity of inducing antibodies against envelope proteins.

**FIG 3:**
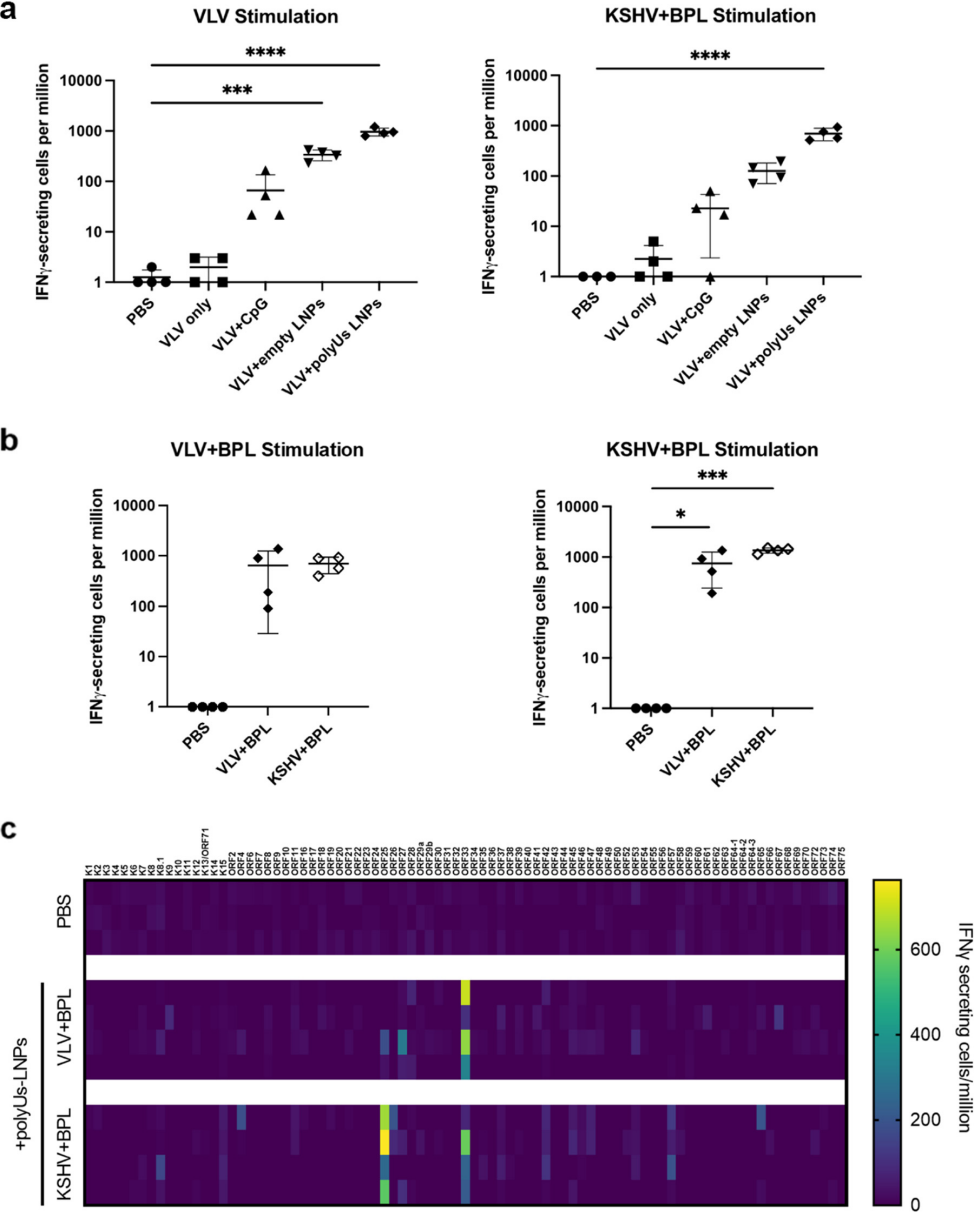
Adjuvanted VLV immunization generated virus-specific T cells. (a) Mice were immunized as described for Fig. 2a. Data show IFN-γ-secreting cell frequencies of VLV-immunized mouse splenocytes stimulated with VLV or KSHV+BPL. (b and c) Mice were immunized as described for Fig. 2d. IFN-γ-secreting cell frequencies of immunized mouse splenocytes stimulated with VLV+BPL or KSHV+BPL were determined (b), and some of the mock-immunized groups (PBS) that gave no IFN-γ-secreting cells and were set as 1 for graphing purposes. For the heatmap showing IFN-γ-secreting cell frequencies from VLV-immunized mouse splenocytes stimulated with overlapping peptide libraries of KSHV ORFs (c), each row represents a different mouse. Raw data are provided in the supplemental material. Statistical analysis for panels a and b entailed an ordinary one-way ANOVA with Dunnett’s test for multiple comparisons to the PBS mock-immunized group (*, *P* < 0.05; **, *P* < 0.01; ***, *P* < 0.001; ****, *P* < 0.0001). Means and standard deviations are shown (*N* = 4 mice per group).

To measure T cell responses after immunization, we performed interferon gamma (IFN-γ) enzyme-linked immunosorbent spot (ELISpot) assays with splenocytes isolated from immunized mice. Cells were stimulated with VLVs or inactivated KSHV virions, and IFN-γ-producing cells responding to stimulation were quantified. VLVs without adjuvant did not elicit any responding T cells. However, significant numbers of IFN-γ-producing cells were observed in mice that received adjuvanted immunizations, with the strongest response coming from mice immunized with VLVs adjuvanted with poly(U)s-LNP (Fig. 3a). In addition, VLV induced a comparable level of cellular immunity to inactivated virions when adjuvanted with poly(U)s-LNP (Fig. 3b). We also used an activation-induced marker (AIM) assay to examine T cell responses. The AIM assay measures the activation of antigen-specific T cells based on the upregulation of the markers CD69 and 4-1BB for CD8 T cells and OX40 and 4-1BB for CD4 T cells. They have been used to detect T cells generated by both infection and vaccination (25–27). Splenocytes from immunized mice (Fig. 2d) were stimulated with VLV+BPL or KSHV+BPL and analyzed by flow cytometry for the expression of activation markers (Fig. S3a). AIM^+^ CD4 and CD8 T cells were detected in mice immunized with VLVs or inactivated KSHV virions (Fig. S3b and c). Moreover, both types of immunogens induced similar levels of AIM^+^ T cells in mice.

To determine which viral proteins were targets for T cell responses, we conducted a proteome-wide ELISpot assay using overlapping peptide libraries derived from viral antigens. In general, T cell responses were heterogeneous among individual mice. However, ORF33, a viral tegument protein, was most frequently recognized by T cells in VLV-immunized mice and was also one of the two frequent targets in mice immunized with inactivated KSHV virions. Another commonly recognized T cell target from inactivated virion immunization was ORF25, the major capsid protein (Fig. 3c). Overall, these data indicated that adjuvanted VLVs can generate KSHV-specific T cell responses.

### VLV immune serum contains neutralizing antibodies.

The major correlate of protection for most commercial vaccines is generally thought to be neutralizing antibodies, because these antibodies can block viral attachment or entry to stop initiation of infection. K8.1 mediates viral attachment by binding heparan sulfate moieties (28, 29), and it is a major target for the VLV immune sera. Notably, we also saw a strong antibody response to ORF4, a complement control protein that also possesses heparan sulfate binding activity (17, 30). In addition, we detected a weak response to gB, which mediates fusion between the viral envelope and the host’s cellular membrane (31). To determine whether VLV-induced antibodies block attachment and entry, we conducted a neutralization assay based on GFP expression in infected cells. We utilized two methods of infection to study viral neutralization: free virus and spin infection. While free virus infection only requires incubation of virus with cells, spin infection is performed by centrifuging virus onto cells and has been used to increased infection efficiency of viruses, including KSHV. Multiple mechanisms, such as centrifugation-induced changes in cellular structure and the facilitation of attachment of virions to cells, have been shown to account for the increase in retrovirus infectivity resulting from spin infection (32, 33). Although KSHV-specific antibodies were significantly increased by coadministration of adjuvants with VLV (Fig. 2a), they possessed modest neutralizing activities (Fig. S4a).

### Complement-enhanced neutralization by VLV immune serum depends on ORF4-SCR-targeting antibodies.

**FIG 4:**
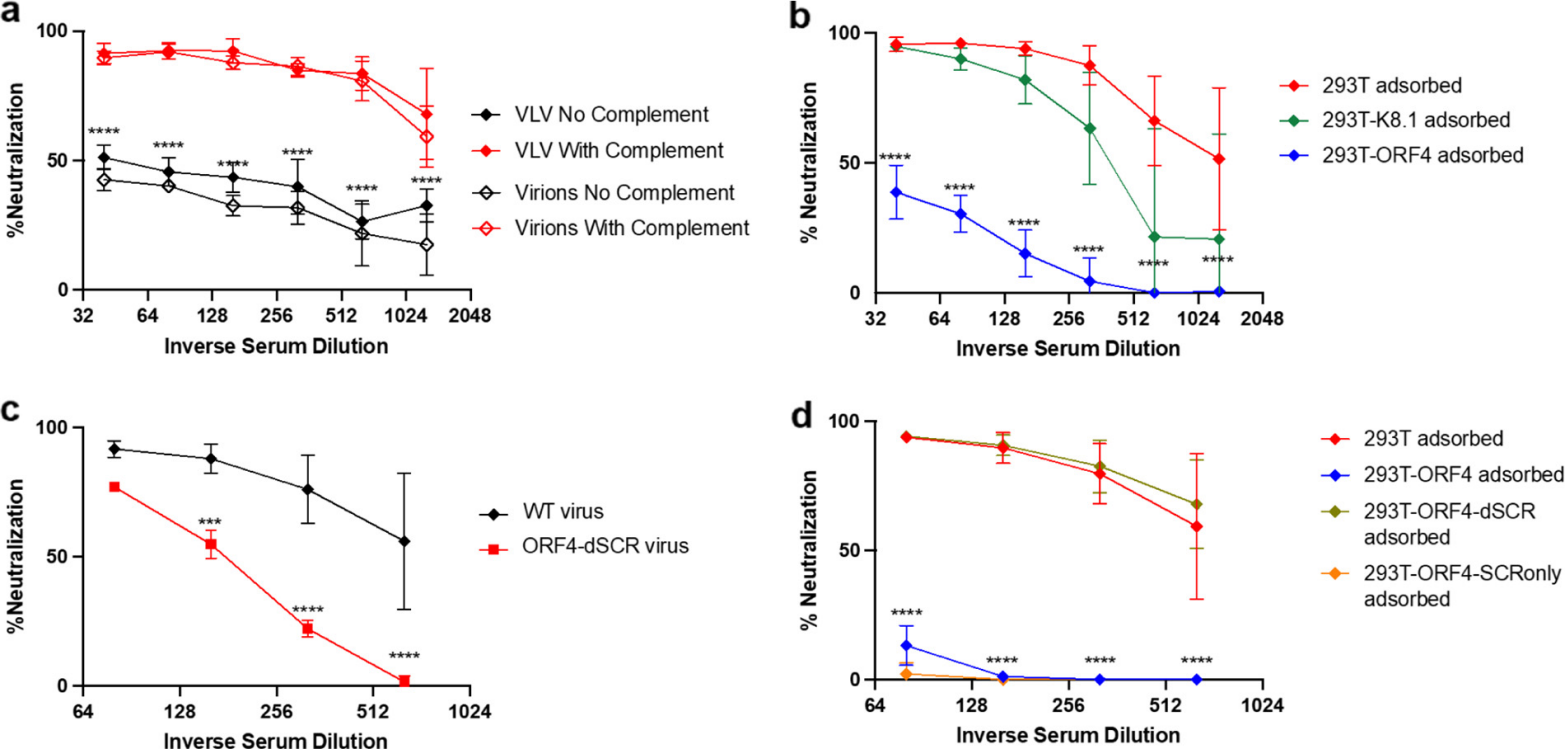
Complement-mediated enhancement of neutralization by VLV-immune serum depended on antibodies targeting ORF4. The immune sera collected at the endpoint in Fig. 2d were serially diluted for neutralization. (a) Spin infection neutralization by the sera of mice immunized with BPL-treated VLV or KSHV virions in the presence or absence of complement. (b) Complement-mediated spin infection neutralization by VLV-immune serum adsorbed on 293T cells or 293T cells expressing K8.1 or ORF4. (c) Complement-mediated spin infection neutralization by VLV-immune serum using WT or ORF4-dSCR virus. (d) Complement-mediated spin infection neutralization by VLV-immune serum adsorbed on 293T cells or 293T cells expressing ORF4, ORF4-dSCR, or ORF4-SCRonly. Neutralization was calculated by comparing the level of GFP^+^ cells to the level obtained from PBS-immune serum (see Materials and Methods for details). Statistical analysis used a two-way ANOVA with Tukey’s multiple-comparison test for differences between responses with and without complement (a), between 293T-adsorbed and 293T-ORF4 adsorbed samples (b), VLV immune sera neutralization of WT and ORF4-dSCR virus (c), or between 293T-adsorbed and 293T-ORF4-SCRonly adsorbed samples (d). Means and standard deviations are shown (*N* = 3 to 4 mice per group).

ORF4, a viral glycoprotein with heparan sulfate binding and complement inhibition activity (34, 35), is one major target of VLV immune serum. Serum containing anti-ORF4 antibodies has been demonstrated to antagonize its complement inhibition function (36). Thus, we hypothesized that the anti-ORF4 antibodies elicited by VLVs could make KSHV susceptible to neutralization by the complement system. To test this, we performed spin infection neutralization assays using heat-inactivated sera from mice immunized with VLV+BPL or KSHV+BPL (Fig. 2d), with or without guinea pig serum (GPS) as a source of complement, due to the instability of mouse complement (37). We used spin infection to keep neutralization minimal without complement. Complement activation can be antibody dependent (classical) or independent (lectin and alternative pathways) (38, 39). Incubating KSHV virions with GPS in the presence of mock immune serum did not result in any reduction in GFP-positive cells (Fig. S4b). However, when the sera from VLV or inactivated virion immunization were included with GPS, we observed a significant increase in neutralization (Fig. 4a). This increase was also observed when normal human serum (NHS) was used as a complement source (Fig. S4c). To determine the role of ORF4-specific antibodies in this complement-enhanced neutralization, we developed an adsorption procedure to deplete ORF4 antibodies by incubating the immune serum samples with 293T cells transiently transfected with an ORF4-expressing plasmid. A similar adsorption by incubating with K8.1-expressing cells was carried out to deplete K8.1-specific antibodies. Antibody depletions were confirmed by IFA and ELISA (Fig. S4d and e). Depletion of anti-ORF4 antibodies caused a significant loss of complement-enhanced neutralization, while depletion of K8.1 antibodies had little impact until higher serum dilutions were used (Fig. 4b). Therefore, ORF4-specific antibodies were critical for complement-enhanced neutralization by the VLV immune serum.

To determine whether ORF4-specific antibodies counteracted the complement-inhibitory function of ORF4 on the viral envelope to enhance complement-mediated neutralization, we generated a viral mutant with a deletion in the SCR region of ORF4 that is responsible for its complement inhibition activity (17). This mutant virus, referred to as KSHV ORF4-dSCR, was expected to be unable to inhibit complement, leading to an increased sensitivity to complement even in the absence of anti-ORF4 antibodies. The expression of ORF4-dSCR was confirmed by Western blotting and IFA analyses (Fig. S5a and b). However, the ORF4-dSCR virus remained resistant to complement (Fig. S5c), indicating that KSHV virions did not activate the complement system in an antibody-independent manner. In the spin infection neutralization assay with the VLV immune sera, the ORF4-dSCR virus was less susceptible to complement-enhanced neutralization than was the WT virus (Fig. 4c). This result did not support the idea that anti-ORF4 antibodies enhance complement activity on KSHV virions in the neutralization assay. Instead, it suggests that the SCR region of ORF4 is needed for the formation of the antibody complex to activate the complement system.

To confirm the importance of SCR binding antibodies in complement-dependent neutralization, we generated plasmids to express the SCR region only (ORF4-SCRonly) or the rest of ORF4 protein (ORF4-dSCR) for antibody depletion. By IFA, we found that ORF4-specific antibodies of the VLV immune serum mainly targeted the SCR region (Fig. S5d). When SCR-specific antibodies were depleted by adsorption on SCR-expressing cells, complement-enhanced neutralization was abolished, whereas the depletion of antibodies binding to the other non-SCR part of ORF4 had no impact (Fig. 4d). Thus, the antibodies binding to the SCR region of ORF4 are key to recruiting and activating the complement system to facilitate neutralization of KSHV virions.

### ORF4-specific antibodies mediate complement deposition on KSHV-infected B cells.

**FIG 5:**
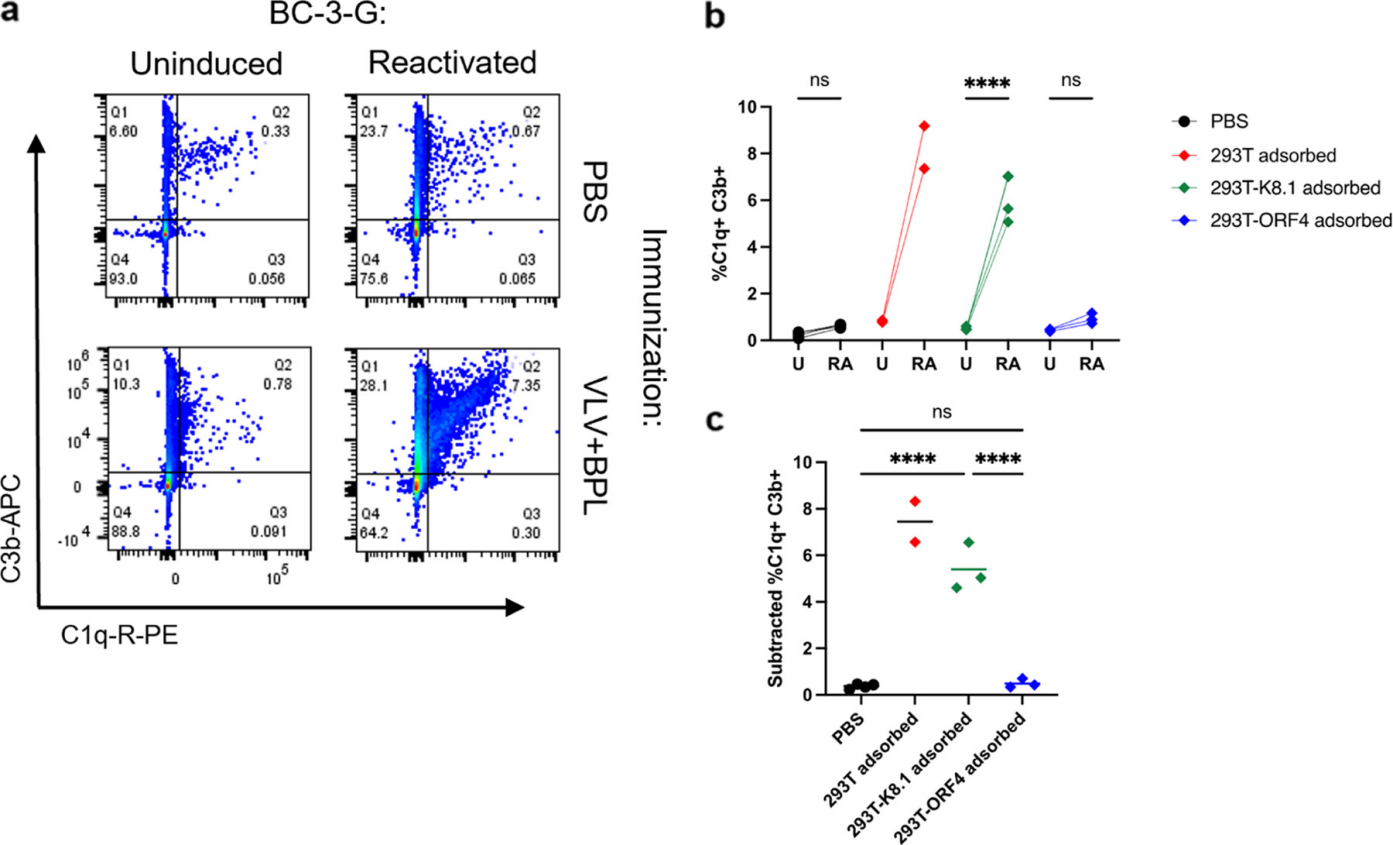
VLV immune serum induces complement deposition on KSHV-infected B cells. (a) Representative flow cytometry plots of BC-3-G cells incubated with VLV immune serum from Fig. 2d and normal human serum and stained for complement deposition. (b) The VLV immune serum adsorbed on various 293T cells and incubated with BC-3G cells, uninduced (UI) or induced to reactivate latent KSHV (RA) in the present of NHS as a complement source. Data are percentages of C1q and C3b double-positive reactivated BC-3-G cells after subtracting background from uninduced BC-3-G cells. (c) Percentages of C1q and C3b double-positive reactivated BC-3-G cells after subtracting background from uninduced BC-3-G cells. Statistical analysis used mixed-effects analysis with Šídák’s test for multiple comparisons (without the 293T-adsorbed group because of *N* = 2) (b) or a one-way ANOVA with Tukey’s test for multiple comparisons without the 293 adsorbed group (because *N* = 2) (c). ****, *P* < 0.0001. Means are shown (*N* = 3 to 4 mice per group).

The classical complement pathway is initiated by the binding of C1q to antibodies and results in the cleavage of component 3 (C3) into C3a and C3b. C3b deposition on the surface of virions and infected cells facilitates their phagocytosis by innate immune cells and may also trigger the assembly of a MAC to destroy them (38). We tested the ability of VLV immune serum to initiate complement deposition with a KSHV-infected B cell line, BC-3-G (40). BC-3-G cells were reactivated to express lytic viral genes, incubated with NHS and VLV immune serum pretreated with different adsorptions, and then analyzed for the binding of complement components C1q and C3b by flow cytometry (Fig. 5a and Fig. S6a). Some adsorbed serum samples produced very high complement binding to uninduced BC3G (Fig. S6b) and were removed from further analysis. The VLV immune serum adsorbed with 293T cells or K8.1-expressing cells could initiate C1q and C3b binding to reactivated BC-3-G cells compared to uninduced cells but not the PBS/mock immune serum or the VLV immune serum adsorbed with ORF4-expressing cells (Fig. 5b). The percentages of increase in double-positive cells upon KSHV reactivation were significantly different between the serum samples depleted of K8.1-specific antibodies and those depleted of ORF4-specific antibodies. No significant difference was found between the ORF4 antibody-depleted serum samples and the mock immune ones (Fig. 5c). Thus, complement deposition on infected B cells depends on ORF4-targeting antibodies.

### ORF4 antibodies in KSHV-seropositive patients lack complement-dependent neutralization.

**FIG 6:**
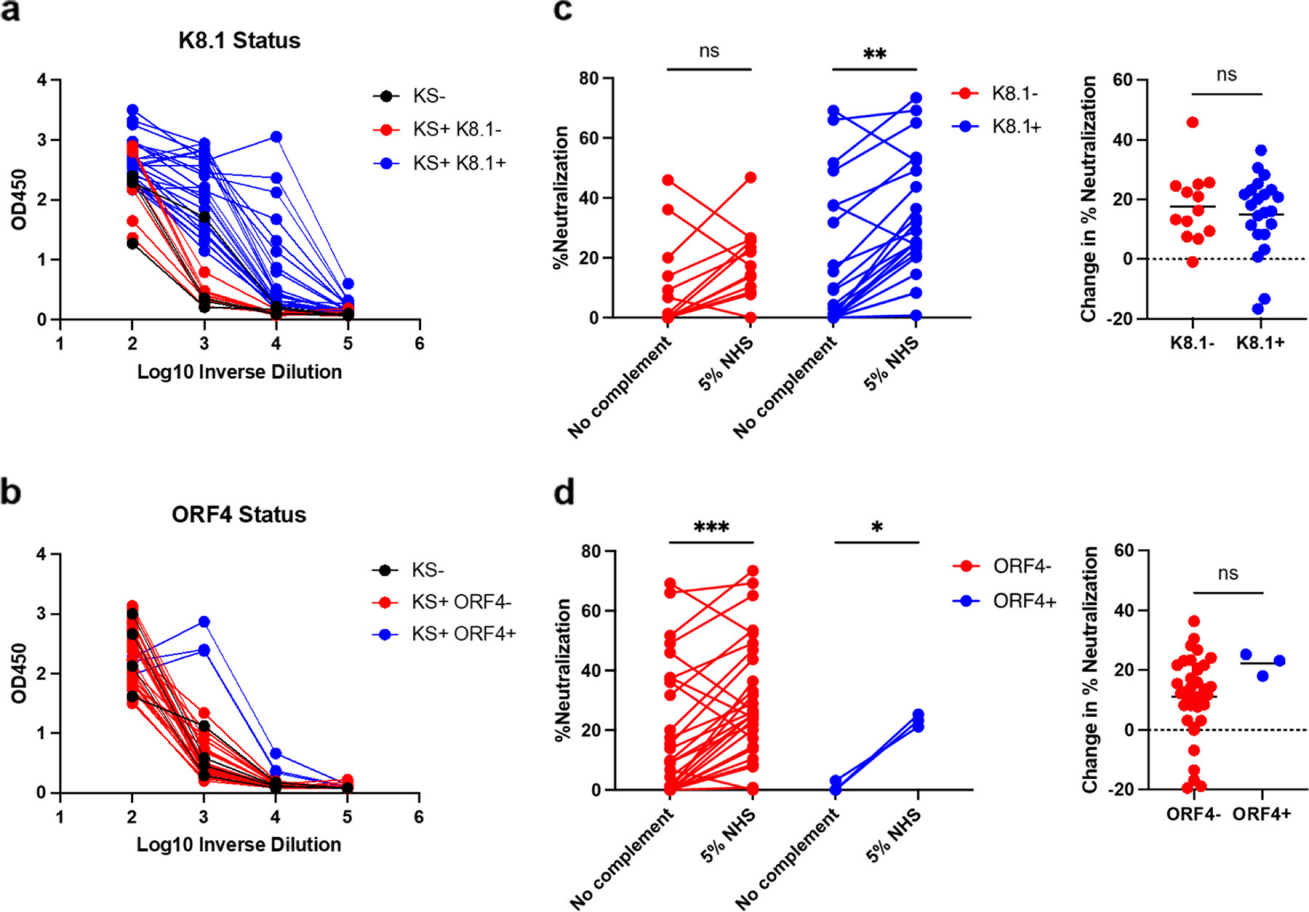
Serum from KS^+^ human patients do not display robust complement-enhanced neutralization. (a and b) K8.1 (a) and ORF4 (b) ELISA signals from serially diluted KS^+^ human serum. (c and d) Neutralization of WT KSHV by serum samples diluted 1:100 in the presence and absence of NHS as a source of complement (left) and the difference in neutralization upon addition of complement (right). Samples are separated by K8.1 (c) or ORF4 (d) serostatus as determined by ELISA. Statistical analysis of data in panels c and d used a mixed-effects analysis with Šídák’s test for multiple comparisons (percent neutralization) or an unpaired *t* test (for change in neutralization). *, *P* < 0.05; **, *P* < 0.01; ***, *P* < 0.001. Means and standard deviations are shown (*N* = 13 K8.1^−^, 26 K8.1^+^, 35 ORF4^−^, or 3 ORF4^+^ human serum samples, including KS^−^ samples).

The contribution of ORF4-specific antibodies in the sera of KSHV-infected individuals to complement-mediated neutralization has not yet been studied (36, 41). K8.1-specific antibodies are the predominant antibodies in infected individuals, while ORF4-specific antibodies are much rarer and lower in quantity (22, 41). We obtained serum samples from patients with and without KS from the Multicenter AIDS Cohort Study (MACS). The KS sera were collected about a year after KS diagnosis. These serum samples were stratified for the presence of K8.1- and ORF4-specific antibodies by ELISA. Of 35 KS^+^ patients, 25 were positive for K8.1 antibodies, but only 3 were positive for ORF4 antibodies, and all 3 were also positive for K8.1 antibodies (Fig. 6a and b). We performed neutralization assays with spin infection on all 35 serum samples at a 1:100 dilution with and without NHS as the source for complement. Only 3 samples reduced the percentage of GFP^+^ cells by more than 50% without complement, and 2 of these samples displayed a moderate increase in neutralization when complement was included. While enhanced neutralization by complement was observed for most samples, only a few achieved over 50% neutralization. When samples were separated by the status of K8.1 or ORF4 antibodies (Fig. 6c and d), those negative for K8.1 antibodies were not significantly affected by including complement in the neutralization assay. This K8.1-negative group likely contained low KSHV-specific antibodies. However, when comparing complement-mediated changes of neutralization, no statistically significant differences were observed between negative and positive statuses of K8.1 or ORF4 antibodies. This was likely attributable to modest complement-mediated changes across all samples. Overall, the sera of KSHV-infected human patients in the UCLA MACS repository have little neutralizing activity and do not display the same robust complement-enhanced neutralization as we observed for the mice sera immunized with VLV or inactivated virions.

## DISCUSSION

Kaposi sarcoma and other KSHV-induced diseases represent a clear need for a prophylactic KSHV vaccine. However, without known correlates of immunity, it is difficult to predict the efficacy of a vaccine candidate. The current preclinical vaccine candidate based on the Newcastle disease virus virus-like particle (VLP) platform is designed to generate neutralizing antibodies against viral attachment and fusion proteins K8.1, gB, and gH/gL to prevent viral infection (42, 43). Our study has shown that that effector functions of antibodies mediated by their Fc region, such as activating complement, can also contribute to protection by neutralizing KSHV virions. In addition, an effective KSHV vaccine should also elicit cellular immunity to eliminate infected cells, because herpesviruses are capable of cell-to-cell transmission that evades neutralization by antibodies (44).

Here, we have presented our results on the immunogenicity of a KSHV VLV-based platform, which incorporates a repertoire of noncapsid viral structural proteins. We demonstrated that coadministration of VLVs with adjuvants elicited comparable cellular and humoral responses to inactivated KSHV virions against antigens shared between virions and VLVs, such as tegument and envelope proteins (Fig. 2 and 3). In addition to a conventional CpG DNA-based adjuvant, we also employed novel LNP-based adjuvants. LNP have recently been shown to be potent adjuvants for protein-based vaccines, and our data support this finding (15, 16, 45). In C57BL/6 mice, we showed that ORF33, a tegument protein, is the major target for T cell responses after VLV immunization, while K8.1- and ORF38-specific antibodies dominate humoral immunity. Moreover, we also observed a strong antibody response toward ORF4, a complement regulatory protein. Although we detected only a low level of neutralizing antibodies, ORF4-targeting antibodies engaged the complement system to neutralize KSHV virions (Fig. 4) and caused complement deposition on infected cells (Fig. 5). These data support the inclusion of ORF4 as an immunogen to engage antibody effector functions in KSHV vaccine development.

Several new classes of adjuvants have been developed to enhance vaccine-induced immune responses (12). Some of these adjuvants stimulate type I interferons (IFN-Is) and proinflammatory cytokines by triggering endosome-located TLRs activated by nucleic acids, such as TLR3, TLR7/8, and TLR9 (12, 46). IFN-Is play an important role in the induction of cellular immunity. TLR7 recognizes GU-rich single-stranded RNA (ssRNA), while TLR9 is activated by unmethylated CpG motifs prevalent in microbial DNA. A CpG-based adjuvant is used in an FDA-approved hepatitis B vaccine (Heplisav-B), and the ssRNA component in the influenza A WIV vaccine is critical for its intrinsic adjuvant activity via TLR7 activation (47). It is worth noting that in the absence of adjuvants, BPL-inactivated KSHV virions were more immunogenic than VLV following intraperitoneal administration (Fig. S1b), presumably due to viral DNA in virions serving as a potential TLR ligand. One major challenge of using RNA as an adjuvant is its instability. The 2019 coronavirus disease (COVID-19) pandemic has brought LNP technology to the forefront of public and scientific thought. *In vivo* delivery of immunostimulatory nucleic acids, such as ssRNA or poly(U)s as TLR7 ligand, can be facilitated by LNP. Moreover, LNP themselves have adjuvant activity from ionizable lipids (16). Further studies will be required to elucidate underlying mechanisms of these adjuvants.

While T cell and antibody responses in KSHV-infected individuals are broad and heterogeneous (22, 48, 49), some viral proteins are more frequently targeted than others. Heterogeneity is not unique to KSHV and is also found for other herpesviruses, such as herpes simplex virus 1 (HSV-1) and HSV-2 (50, 51). This is likely because large viruses like herpesviruses encode many proteins, and variations in processing and presentation of this repertoire among individual hosts result in a spectrum of immune responses. Among the structural proteins shared between VLV and virions, K8.1 and ORF38 are common targets for antibodies in KSHV-infected individuals. Antibody reactivity to these proteins is detected in over 50% of KS patients, although reactivity may be <20% in recently infected children (22, 52). Interestingly, K8.1 and ORF38 reactivity was also observed for the mouse sera following immunization of adjuvanted VLVs and virions (Fig. 2f). Another common target in the immune serum from inactivated virions but not VLVs is ORF65, a capsid protein that is not present in VLVs. ORF65 is also among the three virion-associated proteins identified as common targets for human KS patient serum in addition to K8.1 and ORF38 (22). In contrast, we observed a robust anti-ORF4 antibody response in the sera of mice immunized with both VLV and inactivated virion but not in the sera of the limited number of KS patients we examined (Fig. 6). It has been shown by others that the antibody response to ORF4 is rarely detected in KSHV patients (22, 41). In general, the presence of anti-ORF4 antibodies is associated with higher levels of anti-KSHV antibody responses, which are more common in KS patients. This may be due to the opportunity for less immunogenic viral proteins to stimulate the host immune system during viral reactivation in KS. However, our observation could also be due to a species-specific difference in immunogenicity for ORF4 but not for K8.1, ORF38, or ORF65. In contrast to humoral immunity, we did not observe the same shared T cell targets between mice following immunization compared to humans following infection. For instance, K8.1 is the most frequently recognized T cell target, with K8.1-specific T cells found in up to 30% of KSHV-infected individuals (53), but K8.1-specific cellular responses were undetectable in the immunized mice (Fig. 3). Instead, we identified consistent T cell responses to ORF33 in VLV-immunized mice, while inactivated virion-immunized mice possessed T cells that responded to ORF33 and ORF25. These differences could be due to species-specific peptide presentation, but they could also indicate a difference in antigenicity between immunization and natural infection for certain antigens (54, 55). Interestingly, immunization also generated heterogeneous T cell responses to other viral proteins, which reflected the heterogeneity found in infected individuals (48, 53). A diverse response could be beneficial to optimizing immunity against KSHV, since it is currently unknown which T cell targets would be protective against disease.

VLV immunization generated low levels of neutralizing antibodies despite the delivery of neutralizing targets such as gB and gH/gL (Fig. 1c and Table S1). Although VLV may be a safer alternative to inactivated virions as an immunogen for vaccination, it is less immunogenic than inactivated virions, which have already been shown to elicit a modest level of neutralizing antibodies (42). Thus, other vaccine platforms for generating neutralizing antibodies may need to be pursued. One salient finding is a potential role for complement in KSHV neutralization. The complement cascade is activated by classical (antibody-dependent), lectin, and alternative pathways, converging on the cleavage of C3. C3 cleavage leads to multiple antiviral mechanisms, including the formation of MAC, resulting in the lysis of enveloped viruses and infected cells. In addition to virolysis, complement can neutralize viruses by other mechanisms, such as C3b opsonization of virions to block receptor binding and target virions for phagocytosis (56). KSHV’s ORF4 antagonizes the complement system by reducing C3 cleavage and inactivating C3b (57). Complement inhibition of ORF4 can be blocked by anti-ORF4 monoclonal antibodies or by serum containing anti-ORF4 antibodies (36, 58). However, we found that rather than negating the ability of ORF4 to inhibit complement, ORF4-specific antibodies of the VLV immune sera engaged the complement system to neutralize KSHV virions. Furthermore, we observed that ORF4-specific antibodies mediated deposition of complement onto KSHV-infected B cells. Notably, anti-K8.1 antibodies of the VLV immune sera, while abundant, did not seem to play any significant role in complement-enhanced neutralization (Fig. 5b). The classical pathway is initiated by the binding of C1q to the Fc region of antibodies (56). Because C1q has a weak affinity to a single antibody molecule, it is thought that clustering of antigen-antibody complexes on the surface of cells or virions is necessary for the formation of a multivalent structure for C1q binding and activation. At this moment, it is unclear why K8.1 antibodies cannot afford complement-mediated effector functions. It is possible that the K8.1 antigen-antibody complex is unable to form a high-avidity C1q binding structure. This could be due to the relatively smaller size of K8.1 compared to ORF4 or a difference in arrangement on the viral envelope. It is also unclear whether antibodies against other glycoproteins in addition to ORF4 would be able to stimulate complement activity. Although it is important to consider complement as a protective immune mechanism for KSHV vaccine development, this will still need to be tested in preclinical models.

In contrast with antibodies in VLV-immunized mice, ORF4-targeting antibodies are not commonly detected in the sera of KSHV-infected individuals (22, 41). Two studies have examined the function of anti-ORF4 antibodies in the context of complement. In one study, patient sera that contained ORF4-specific antibodies increased antibody-mediated C3b deposition on ORF4-expressing cells compared to mock-transfected cells, and this increase was not seen with normal human serum (41). However, that study did not distinguish whether this increase was because anti-ORF4 antibodies blocked ORF4 function or because these antibodies activated complement through the classic pathway. In a follow-up study, ORF4-mediated C3b degradation was inhibited by the patient serum sample that contained the highest level of ORF4 antibodies (36). Neither of those two studies examined complement-mediated antiviral effects on KSHV. In the current study, we attempted to characterize the role of human ORF4-specific antibodies in complement-enhanced neutralization. Among 35 KS^+^ human serum samples obtained from the UCLA MACS repository, only 2 samples, both positive for K8.1, were able to neutralize KSHV infectivity by over 50% at a 1:100 dilution. This was in contrast with previous findings that certain individuals with KS possessed high levels of neutralizing antibodies (59, 60). Only 3 of the 35 UCLA samples were positive for ORF4-specific antibodies, and thus, it was difficult to reach a statistically significant conclusion on whether the presence of these antibodies can strongly enhance complement-mediated neutralization. Future studies on large cohorts with a diverse KSHV infection status will be needed to study the role of ORF4 antibodies. Considering that humoral responses to KSHV infection in humans are focused on anti-K8.1 antibodies, it was not surprising to see modest complement-mediated enhancement of neutralization from human serum samples, which was consistent with our data that anti-K8.1 antibodies in VLV immune sera also do not play a major role in complement-mediated neutralization.

Complement is an important part of the host innate immune defense against pathogens and, naturally, viruses have evolved multiple strategies to counteract the complement system. Gammaherpesviruses, like KSHV and murine gammaherpesvirus-68 (MHV-68), encode homologues of host regulators of complement activation (RCAs), like ORF4. Alphaherpesviruses, like HSV-1 and HSV-2, also encode a viral RCA, glycoprotein C (gC). The lack of functional gC makes HSV-1 highly sensitive to complement-mediated neutralization in both the presence as well as the absence of anti-HSV antibodies (61). Anti-gC antibodies elicited by immunization block gC’s complement inhibition and significantly increase the neutralization effectiveness of antibodies against gD, the receptor binding protein of HSV, in the presence of complement (62). Therefore, adding gC to gD for immunization enhances the efficacy and protection afforded by multiple vaccine formulations (62–65). This supports the role of complement in vaccine-mediated protection for HSV-1 and HSV-2. Another herpesvirus for which complement may play a significant role in vaccine responses is human cytomegalovirus (hCMV). gB is the fusion protein and immunodominant antibody target in hCMV-infected individuals (66). In clinical trials, a gB vaccine conferred 50% efficacy against hCMV acquisition in young women, despite low neutralizing titers in the sera without complement (67, 68). It has been suggested that Fc-mediated effector functions of antibodies are critical for the observed efficacy (69). Complement-enhanced neutralization was detected in the sera from people who received a gB vaccine, suggesting that complement may be one of the protective mechanisms (68, 70). Notably, ORF4 has been demonstrated to be a virulence factor in the MHV-68 model during acute replication, but it is not necessary for latency establishment (71). However, the impact on long-term latency is unclear, as ORF4 may be needed during reactivation, which maintains the pool of latently infected cells. Antibodies against ORF4 reduced MHV-68 latency in B cell-deficient mice, where persistent lytic viral replication takes place (72). Thus, the complement system is important for controlling herpesvirus pathogenesis, and targeting viral inhibitors of complement will likely play a key part in effective vaccines.

In conclusion, our study showed that VLV in the current format is not an effective immunogen to elicit neutralizing antibodies. Nonetheless, ORF4-specific antibodies of the VLV immune sera activate complement to neutralize KSHV virions, supporting the consideration of eliciting antibodies that possess effector functions in developing KSHV vaccines.

### Limitations of this study.

The experiments presented here have examined the short-term immune response after adjuvanted VLV immunization and demonstrated the importance of engaging the complement system in these responses. Despite the small experimental group sizes at 3 to 4 mice per group, multiple immunizations with different batches of VLVs were performed to confirm the reproducibility of our main conclusions. Our study initially aimed to investigate KSHV VLV as a safe vaccine platform. However, we still detected a trace amount of viral DNA and possible infectious units in the VLV sample. Although we found no evidence of successful infection from KSHV VLV, the risk will still need to be carefully assessed. There are also other aspects to evaluate before further development of a VLV vaccine. For example, the scalability and stability of VLVs will need to be assessed. Regardless of whether VLVs will be further developed into a clinical KSHV vaccine, our work suggests a new antibody-mediated mechanism to control KSHV infection.

## MATERIALS AND METHODS

### Plasmids and cell lines.

KSHV glycoprotein sequences were amplified from KSHV BAC16 derived from rKSHV.219 (a gift from Jae Jung) (21). Sequences were cloned into the pCAG-GFPd2 overexpression vector (a gift from Connie Cepko, Addgene, Watertown, MA; catalog number 14760) using the EcoRI and BglII cut sites to replace the GFP coding region or into the pcDNA3-OVA vector (a gift from Sandra Diebold and Martin Zenke; Addgene catalog number 64599), replacing the OVA coding region. Severe acute respiratory syndrome coronavirus 2 (SARS-CoV-2) Spike was expressed from pCMV14-3X-FLAG-SARS-CoV-2 S (a gift from Zhaohui Qian; Addgene catalog number 145780) as a negative control. K8.1 and ORF4 were cloned with FLAG tags on the extracellular domain right after the signal sequence (after amino acid 28 for K8.1 and after amino acid 20 for ORF4). ORF4 without the SCR region was cloned by removing amino acids 24 to 314 from the original protein sequence. The SCRonly version of ORF4 was cloned by removing amino acids 315 to 519 and connecting the SCR region to the transmembrane domain of ORF4 with a GGGGS linker. gB was cloned with a FLAG tag on the C-terminal end. C-terminal FLAG-tagged gL was connected to C-terminal hemagglutinin (HA)-tagged gH with a P2A sequence for stoichiometric coexpression and proper association of the gH/gL complex (73). Signal peptides were determined using SignalP version 4.1 (74, 75), and transmembrane regions were determined using TMHMM-2.0 (76). Cloning primers were synthesized by Integrated DNA Technologies (IDT; Coralville, IA), and plasmids were confirmed via Sanger sequencing by Laragen, Inc. (Culver City, CA).

293T and HEK293 cells were cultured in Dulbecco’s modification of Eagle’s medium (DMEM) (Corning catalog number 10017CV; Corning, NY) supplemented with 10% fetal bovine serum (FBS; Corning catalog number 35010CV) and 1× penicillin-streptomycin (Corning catalog number 35010CV). iSLK cells containing latent KSHV were cultured in DMEM supplemented with 10% FBS and 1× penicillin-streptomycin with 1 µg/mL puromycin, 250 µg/mL G418, and 1,200 µg/mL hygromycin (all from Invivogen, San Diego, CA; catalog numbers ant-pr-1, ant-gn-2, and ant-hg-5) for selection. iSLK-WT cells harboring BAC-16 that produced GFP-expressing KSHV were a gift from Jae Jung (21). iSLK-25Δ60 cells were generated as previously described (18).

KSHV ORF4-dSCR was generated with *en passant* mutagenesis to replace amino acids 24 to 314 of ORF4 with the HA tag using GS1783 Escherichia coli in accordance with previously described protocols (77). BAC DNA containing the KSHV ORF4-dSCR genome was analyzed by restriction enzyme digestion to ensure no gross rearrangements occurred and was transfected into 293T cells using Lipofectamine (Thermo Fisher Scientific, Waltham, MA; catalog number 11668027). 293T cells stably harboring the viral genome were selected with 100 μg hygromycin. 293T cells were cocultured with iSLK cells in the presence of 20 ng/mL 12-*O*-tetradecanoyl-phorbol-13-acetate (Fisher Scientific, Waltham, MA; catalog number AAJ63916MCR) and 1 mM sodium butyrate (Fisher Scientific; catalog number AC263191000) to induce infection of iSLK cells. Infected iSLK cells were selected with iSLK cell medium described above to establish iSLK-ORF4-dSCR. Replacement of the SCR region with the HA tag was confirmed by Western blotting on lysates from reactivated iSLK-ORF4-dSCR cells and by IFA (Fig. S5a and b).

### Virus production, isolation, and inactivation.

Wild-type KSHV, KSHV-ORF4dSCR, and VLV were produced by reactivating the latent KSHV virus in iSLK-WT, iSLK-ORF4dSCR, and iSLK-25Δ60 cells, respectively, with DMEM containing 10% FBS, 1× penicillin-streptomycin, 5 µg/mL doxycycline, and 1 mM sodium butyrate. Supernatants were harvested after 4 to 5 days of reactivation, when >90% of cells exhibited cytopathic effect.

For virus used for neutralization assays, supernatants collected from 4 to 6 10-cm dishes of reactivated iSLK cells were clarified at 2,000 × *g* for 10 min, and the virus was concentrated at 21,000 × *g* for 90 min. Pellets were washed gently with serum-free DMEM before resuspension in serum-free DMEM. The virus suspension was clarified to remove debris by centrifugation at 7,000 rpm for 3 min in a microcentrifuge.

For VLVs and virions used for immunization studies and T cell stimulation, supernatants collected from 20 to 30 15-cm dishes of reactivated iSLK cells were clarified at 8,000 × *g* for 10 min. Virions were pelleted by ultracentrifugation at 80,000 × *g* for 1 h in sterile ultracentrifuge tubes (Beckman Coulter, Indianapolis, IN; catalog number C14292), and the pellets were incubated in Dulbecco’s phosphate-buffered saline (DPBS; Thermo Fisher Scientific catalog number 14190250) overnight. The resuspended viral pellets were then loaded on top of a sucrose cushion consisting of 25% sucrose in DPBS over 50% sucrose in DPBS and centrifuged at 100,000 × *g* for 1 h in sterile ultracentrifuge tubes (Beckman Coulter catalog number C14297). The band at the sucrose interface was collected, diluted in DPBS, and centrifuged at 80,000 × *g* for 1 h. The final pellet was incubated in DPBS overnight before resuspension. Preparations were separated into single-use aliquots immediately after resuspension and stored at −80°C. Viral protein concentration was determined by standard Bradford assay with bovine serum albumin (BSA) as the standard (Fisher Scientific catalog number BP9703100).

For chemical inactivation, KSHV or VLV was resuspended in 1 mL DPBS containing 100 mM sodium phosphate (Fisher Scientific catalog number S374-500). One microliter of BPL (Alfa Aesar, Haverhill, MA; catalog number AAB2319703) was added for a final concentration of 0.1% (vol/vol). This solution was inverted at room temperature overnight to inactivate virus. BPL was removed by dialysis against DPBS at 4°C overnight using a 15-kDa molecular weight cutoff Tube-O-Dialyzer (G-Biosciences, St. Louis, MO; catalog number 786618). Dialyzed preparations were collected, separated into single-use aliquots, and stored at −80°C.

### Cryo-electron microscopy.

Aliquots of 2.5 μL of VLV and virion preparations were applied to glow-discharged Quantifoil 200-mesh Cu R2/1 grids (Electron Microscopy Sciences, Hatfield, PA; catalog number Q250CR1). Grids were plunge-frozen in liquid ethane using an FEI Vitrobot Mark IV at 22°C and 100% humidity. Images were acquired at 25,000× and 50,000× on an FEI Tecnai TF20 equipped with a 4k-by- 4k TVIPS F415MP charge-coupled-device detector.

### Immunogold staining.

Aliquots of 2.5 μL of VLV or virion preparations were incubated on glow-discharged homemade 200-mesh Formvar/carbon-coated copper grids for 5 min at room temperature. After sample incubation, the grids were passed through two drops of blocking buffer (PBS plus 0.4% BSA, filtered) on Parafilm and floated on a third drop for 30 min in a homemade moisture chamber. Excess blocking buffer was removed by lightly blotting with filter paper prior to incubation with primary antibody against K8.1 (Santa Cruz Biotechnology, Dallas, TX; catalog number sc-65446) diluted 1:50 in blocking buffer for 1 h. Negative controls substituted primary anti-K8.1 with anti-FLAG M2 (Sigma-Aldrich, St. Louis, MO; catalog number F1804) or blocking buffer. After primary antibody incubation, the grids were washed by passing through two drops of blocking buffer and floating on a third drop for 10 min. The washed grids were lightly blotted before incubation with 6-nm gold-conjugated Fabs of goat anti-mouse IgG (Electron Microscopy Sciences; catalog number 25374) diluted 1:20 in blocking buffer for 1 h. The grids were washed with three drops of PBS and kept floating on a drop of PBS for at most 1 h until ready for negative staining. For negative staining, grids were washed with three drops of distilled water and stained with 2% uranyl acetate (Electron Microscopy Sciences; catalog number 22400-2) for 1 min. Particles that were >100 nm in diameter and had five or more gold beads were considered positively labeled.

### Mass spectrometry.

For MS, 20 μL of each VLV or KSHV preparation was diluted with 80 μL of a master mix consisting of 43 μL HPLC water, 25 μL 8 M urea, 10 μL 100 mM ammonium bicarbonate, and 1 μL 100 mM dithiothreitol (DTT). Samples were then incubated at 60°C to reduce disulfide linkages for 30 min. Iodoacetamide (IAA) was then added to a 10 mM final concentration to alkylate free cysteines, and lysates were incubated in the dark at room temperature for 30 min. Lysates were next digested with Trypsin Gold (Promega Corporation, Madison, WI; catalog number V5280). Trypsin (0.4 μg) was added to each sample, and lysates were then incubated for 16 h at 37°C while being vortexed at 1,000 rpm. Trypsin activity was quenched by adding 10% (vol/vol) trifluoroacetic acid (TFA) to a final concentration of 0.1% TFA. Samples were then desalted on a C_18_ minispin column (The Nest Group, Inc., Ipswich, MA; catalog number HUM S18V) per the manufacturer’s protocol. Samples were eluted from these columns with 200 µL 40% acetonitrile (ACN)–0.1% TFA. Samples were dried by vacuum centrifugation and stored at −80°C until analysis.

All samples were analyzed on an Orbitrap Eclipse mass spectrometry system equipped with an Easy nLC 1200 ultrahigh-pressure liquid chromatography system (Thermo Fisher Scientific) interfaced via a Nanospray Flex nanoelectrospray source. Immediately prior to analysis, lyophilized samples were resuspended in 0.1% formic acid (FA). Samples were injected on a C_18_ reverse-phase column (30 cm by 75 μm [inner diameter]) packed with ReprosilPur 1.9-μm particles. Mobile phase A consisted of 0.1% FA, and mobile phase B consisted of 0.1% FA–80% ACN. Peptides were separated by an organic gradient from 5% to 35% mobile phase B over 120 min, followed by an increase to 100% B over 10 min at a flow rate of 300 nL/min. Analytical columns were equilibrated with 3 μL of mobile phase A. To build a spectral library, individual samples were analyzed by a data-dependent acquisition (DDA) method. DDA data were collected by acquiring a full scan over a *m/z* range of 375 to 1025 in the Orbitrap at 120,000-resolution resolving power (200 *m/z*) with a normalized AGC target of 100%, an RF lens setting of 30%, and an instrument-controlled ion injection time. Dynamic exclusion was set to 30 s, with a 10-ppm exclusion width setting. Peptides with charge states of 2 to 6 were selected for tandem MS (MS/MS) interrogation using higher-energy collisional dissociation (HCD) with a normalized HCD collision energy of 28%, with 3 s of MS/MS scans per cycle. All individual samples were analyzed by a data-independent acquisition (DIA) method. DIA was performed on all individual samples. An MS scan was performed at 60,000 resolution (200 *m/z*) over a scan range of 390 to 1010 *m/z*, an instrument-controlled AGC target, an RF lens setting of 30%, and an instrument-controlled maximum injection time, followed by DIA scans using 8-*m/z* isolation windows over 400 to 1000 *m/z* at a normalized HCD collision energy of 28%.

Spectral libraries were built with Spectronaut factory settings from DDA pools and from DDA runs from a previous SARS-CoV-2 study (78). Individual samples run with DIA methods were then analyzed against the before-mentioned library with Spectronaut as previously described (79). False-discovery rates were estimated using a decoy database strategy (80). All data were filtered to achieve a false-discovery rate of 0.01 for peptide-spectrum matches, peptide identifications, and protein identifications. Search parameters included a fixed modification for carbamidomethyl cysteine and variable modifications for N-terminal protein acetylation and methionine oxidation. All other search parameters were defaults for the respective algorithms. Analysis of protein expression was conducted utilizing the MSstats statistical package in R. Output data from Spectronaut was annotated based on the human reference (Swiss-Prot human-reviewed sequences downloaded on 10 October 2019) and the human herpesvirus-8 BAC16 strain (sequences were extracted from NCBI GenBank accession number GQ994935.1 on 15 March 2022). Technical and biological replicates were integrated to estimate log_2_ fold changes, *P* values, and adjusted *P* values. All data were normalized by equalizing median intensities, the summary method was Tukey’s median polish, and the maximum quantile for deciding censored missing values was 0.999. Significantly dysregulated proteins were defined as those which had a fold change value of >2 or <(−2), with a *P* value of <0.05.

### Viral DNA extraction and RT-PCR.

DNA extraction was performed using the DNeasy blood and tissue kit (Qiagen, Germantown, MD; catalog number 69504). One microgram of protein from VLV or KSHV preparations was mixed with 10 µg salmon sperm DNA (Thermo Fisher Scientific; catalog number 15632011) as a carrier. Eluted DNA was adjusted to 100 ng/μL, and 1 μL was used for reverse transcription-PCR (RT-PCR). RT-PCR was performed using a 10-μL reaction volume of the iTaq Universal SYBR green supermix (Bio-Rad, Hercules, CA; catalog number 1725120) with primers specific for the ORF50 gene (F, 5′-CGCAATGCGTTACGTTGTTG-3′; R, 5′-GCCCGGACTGTTGAATCG-3′) (81) on a 96-well CFX Connect real-time PCR system (Bio-Rad). Cycle threshold (*C_T_*) was compared to the threshold obtained using primers for the actin gene (F, 5′-CATGTACGTTGCTATCCAGGC-3′; R, 5′-CTCCTTAATGTCACGCACGAT-3′) to normalize to the amount of carrier DNA isolated. THe ORF50/actin ratio was calculated using the formula 2[C*_T_*(actin) − C*_T_*(ORF50)]^−1^.

### KSHV titer and neutralization assay.

KSHV viral titer was obtained by infecting 10,000 HEK293 cells seeded overnight in a 96-well plate. A 50-μL aliquot of virus diluted in serum-free DMEM was added to each well and incubated at 37°C for 2 h. For spin infection, an additional 50 μL of serum-free DMEM was added to each well, and the plate was spun at 400 × *g* for 20 min before the 2-h incubation. The inoculum was removed and replaced with growth medium, and the mixture was incubated for an additional 2 days. Infection was measured by flow cytometry for GFP^+^ cells on an Attune NxT system with a plate autosampler (Thermo Fisher Scientific). Flow cytometry data were analyzed with FlowJo (BD, Ashland, OR).

KSHV neutralization was performed by infecting 10,000 HEK293 cells seeded overnight in a 96-well plate. The amount of virus used was calculated to give ~5% GFP^+^ cells. This fixed amount of KSHV virions was mixed with serially diluted serum samples from immunized mice for 1 h at 37°C before addition onto HEK293 cells. For spin infection, a smaller amount of KSHV virion was used to give ~5% GFP-positive cells, due to the enhanced rate of infection. With the same amount of virions, spin infection resulted in ~3 times more GFP^+^ cells than free virus infection.

All assays comparing complement-enhanced neutralization were performed using spin infection. Immune serum was diluted in DMEM containing 2% heat-inactivated normal mouse serum (Abcam, Cambridge, UK; catalog number ab7486), and KSHV was diluted in serum-free DMEM. For complement-dependent neutralization, KSHV was diluted with DMEM containing 2% GPS or 10% NHS (Complement Technology Inc., Tyler, TX). Diluted serum was mixed with diluted virus at a 1:1 ratio and incubated at 37°C for 1 h. A 50-μL volume of the serum-virus mixture was used to infect cells as described above for the viral titer determinations.

Neutralization was calculated as follows: (% GFP_PBS_ − % GFP_immune_)/(% GFP_PBS_ × 100), where % GFP_PBS_ is the percentage of cells expressing GFP from wells infected with virus mixed with control serum from mock-immunized mice and % GFP_immune_ is from experimental wells infected with virus mixed with serum from immunized mice. If % GFP_immune_ was greater than % GFP_PBS_, neutralization was defined to be zero. The 50% neutralization titer was determined by the last dilution of immune serum that gave more than a 50% reduction in GFP-positive cells compared to the serum samples from mock-immunized mice.

### Mice and immunizations.

Six- to 10-week-old female C57BL/6J mice (The Jackson Laboratory, Bar Harbor, ME; catalog number 000664) were immunized with PBS, VLV, or KSHV virions in a 50-μL volume intramuscularly or a 200-μL volume intraperitoneally using insulin syringes (Becton, Dickinson, and Company [BD], Franklin Lakes, NJ; catalog number 329461) at the described time points. Immunogens were premixed with adjuvants and injected in the same volume described above. ODN2395 CpG adjuvant was purchased from Invivogen (catalog number vac-2395-1). The 21-mer poly(U) with phosphothioate linkages [poly(U)s] was custom synthesized by IDT. Lipid nanoparticles were prepared by Acuitas Therapeutics using a self-assembly process as previously described (82); the ionizable cationic lipid and LNP composition are described elsewhere (83). At the experimental endpoint, mice were euthanized and blood was collected by cardiac puncture with tuberculin syringes (BD catalog number 309623). Serum was collected by centrifugation in serum gel tubes (Sarstedt, Numbrecht, Germany; catalog number 41.1378.005) and heat inactivated at 56°C for 30 min before storage at −80°C. Splenocytes were harvested in RPMI (Corning catalog number 10040CV) containing 10% FBS and 1× penicillin-streptomycin. Single-cell suspensions were prepared by pushing spleens through 70-μm cell strainers (Fisher Scientific catalog number 22-363-548). Red blood cells were removed with ACK lysing buffer (Thermo Fisher Scientific catalog number A1049201). Splenocytes were resuspended in complete RPMI and stored at 4°C for no longer than overnight until stimulation.

### Ethics statement.

All animal experiments were conducted with the approval of the UCLA Institutional Animal Care and Use Committee and the Chancellor’s Animal Research Committee.

### Bead-based multiplexed KSHV antibody assay.

The bead-based multiplex assay was adapted to murine antibodies from a previously described protocol (22). Briefly, 68 recombinant KSHV antigens were each covalently attached to Bio-Plex Pro Magnetic COOH beads (Bio-Rad catalog numbers MC10026 to MC10065) via a sulfo-*N*-hydroxysulfosuccinimide-mediated ester according to the manufacturer's protocol. Antigens were split into two sets, with K8.1, ORF38, and ORF73 present in both sets to ensure consistency. A total of 2,500 beads in 50 µL of assay buffer were added to each well, and 50 µL of serum diluted 1:200 was added. Beads were incubated with serum for 1 h at room temperature and washed with assay buffer. Beads were then incubated with an R-phycoerythrin (PE)-labeled goat anti-mouse IgG secondary antibody (Jackson ImmunoResearch Laboratories, Inc., West Grove, PA; catalog number 115-116-146) for 30 min. Samples were washed, resuspended in 100 µL assay buffer, and analyzed on the Bio-Plex 200 system (Bio-Rad catalog number 171000201). The median fluorescence intensity across all counted beads was computed for each sample and recorded after subtracting the background fluorescence.

### ELISpot assay.

ELISpot using VLV and KSHV+BPL stimulation was performed using a murine TNF-α, IFN-γ ELISpot kit (Cellular Technology Limited [CTL], Shaker Heights, OH; catalog number mIFNgTNFa-1 M/10) following the manufacturer’s instructions. Negative-control wells contained unstimulated splenocytes and positive-control wells were stimulated with a cocktail of phorbol 12-myristate 13-acetate and ionomycin (Thermo Fisher Scientific catalog number 00-4970-03). Experimental wells were stimulated with 1 μg/mL VLV, VLV+BPL, or KSHV+BPL as indicated. A total of 500,000 cells were used per well and plates were incubated for 20 to 22 h at 37°C before development. Plates were scanned and spot counts were analyzed by CTL. The number of spots in negative-control wells was subtracted from values for experimental wells to determine the number of antigen-specific spot-forming cells.

### Activation-induced marker assay.

For the AIM assay, 250,000 splenocytes were plated in 100 μL complete RPMI in a 96-well U-bottom plate (Fisher Scientific catalog number FB012932) and stimulated under the same conditions as for ELISpot. After 18 to 20 h, cells were transferred to a 96-well V-bottom plate (Corning catalog number 3897) and washed with fluorescence-activated cell sorter (FACS) buffer (DPBS containing 2% FBS and 0.05% sodium azide). All wash steps were followed by centrifugation at 500 × *g* for 5 min to pellet cells. Cells were resuspended in 50 µL DPBS containing a 1:25 dilution of Fc block (anti-CD16/CD32 clone 93; Thermo Fisher Scientific catalog number 14-0161-86) and incubated at 4°C for 10 min. Fifty microliters of cell surface antibodies was added to a final dilution of 1:200, and cells were further incubated for 30 to 60 min at 4°C. Cells were stained with the following antibody cocktail: anti-TCR beta-peridinin chlorophyll protein (PerCP)-Cy5.5 clone H57-597, CD4-PE-Cy7 clone RM4-5, CD8a-PE clone 53-6.7, CD69-fluorescein isothiocyanate (FITC) clone H1.2F3, OX40-Super Bright 780 clone OX-86, and 4-1BB-APC clone 17B5 (Thermo Fisher Scientific catalog numbers 50-158-64, 50-154-37, 50-112-9416, 50-965-3, 78-134-182, and 50-112-9043, respectively). After staining, cells were washed twice with FACS buffer and then fixed in 1% paraformaldehyde with incubation at 4°C for 20 min. After fixation, cells were washed twice in FACS buffer and then resuspended in FACS buffer. Cells were analyzed on an Attune NxT with a plate autosampler with single color controls for compensation. Data were analyzed using FlowJo. AIM^+^ cells were defined as OX40^+^ 4-1BB^+^ for CD4 T cells and CD69^+^ 4-1BB^+^ for CD8 T cells. Antigen-specific AIM^+^ cells were calculated by subtracting AIM^+^ cells in unstimulated samples from numbers in stimulated samples.

### KSHV ORFeome ELISpot.

The ORFeome assay was adapted to murine cells from a previously described protocol (48). KSHV 15-mer peptides overlapping by 10 amino acids spanning the entire KSHV genome and totaling over 7,500 were synthesized as previously described (Mimotopes, Victoria, Australia). Peptide sequences were based on the sequence of BC-1 cell line-derived virus. Eighty-five peptide pools were prepared, each corresponding to a single KSHV ORF, except ORF64, which was represented by 3 pools. A total of 83 ORFs were represented. The individual lyophilized peptides were reconstituted using 50% acetonitrile. Peptide pools representing each ORF were prepared by combining the reconstituted peptides corresponding to that ORF. The pools were frozen and relyophilized. The lyophilized pools were reconstituted in dimethyl sulfoxide (<20%) and PBS. A concentration of 5 μg/mL per peptide was used in the assay.

A single 96-well precoated mouse IFN-γ ELISpot plate (Mabtech, Cincinnati, OH; catalog number 3321-4AST-2) was used per mouse to map responses to the 83 KSHV ORFs. Two positive controls, concanavalin A and cell stimulation cocktail (Thermo Fisher Scientific), and one negative control, simian immunodeficiency virus Gag CM9 peptide (New England Peptide, Gardner, MA) were used. VLV was used as a positive control for VLV and KSHV-vaccinated mice and as a negative control for mock-vaccinated mice. A medium-only control was used to monitor background activity. All controls excluding the medium-only well were plated in duplicate, the KSHV ORFs were plated in single wells, and medium-only controls were plated in triplicates. Each well of the ELISpot plate was seeded with 160,000 to 500,000 freshly processed splenocytes and incubated for 18 h at 37°C, 5% CO_2_. Two-step detection with R4-6A2-biotin and streptavidin-alkaline phosphatase, developed with 5-bromo-4-chloro-3-indolylphsophate–nitroblue tetrazolium substrate, was used and plates were read using the CTL ImmunoSpot analyzer.

### K8.1 and ORF4 ELISA.

ELISA plates were prepared by coating with recombinant K8.1 or ORF4 protein as previously described (22). Coated 96-well plates were blocked with assay buffer consisting of DPBS with 2.5% (wt/vol) BSA, 2.5% (vol/vol) normal goat serum (Equitech-Bio, Kerrville, TX; catalog number SG-0500), 0.005% (vol/vol) Tween 20 (Fisher Scientific catalog number BP337-500), and 0.005% (vol/vol) Triton X-100 (Fisher Scientific catalog number BP151-500) and stored at −80°C until use. All serum samples and secondary antibodies were diluted in assay buffer. Coated plates were thawed at room temperature on an orbital shaker and washed with DPBS containing 0.1% (vol/vol) Tween 20 (PBS-T) twice for 3 min. Plates were then washed twice quickly with PBS-T before the addition of 50 μL serially diluted immune serum. Six to eight wells per plate were incubated with assay buffer containing no primary antibody as a background control. Plates were incubated for 1 to 2 h at room temperature on an orbital shaker. Plates were washed with PBS-T twice for 3 min, then twice quickly before the addition of 50 μL 1:4,000 goat anti-mouse horseradish peroxidase secondary antibody (Thermo Fisher Scientific catalog number 62-6520) or 1:5,000 KPL peroxidase-labeled goat anti-human IgG (gamma; LGC Clinical Diagnostics; catalog number 474-1002). Secondary antibody was incubated for 1 h at room temperature with shaking. Plates were then washed once with PBS-T for 3 min, then four times quickly. After one final wash with PBS (no Tween 20), 100 μL 1-Step Ultra TMB ELISA substrate (Thermo Fisher Scientific catalog number 34028) was added to each well. Plates were covered to protect them from light and incubated at room temperature for 30 min with shaking. Signal development was stopped by the addition of 100 μL 1 M sulfuric acid (Sigma-Aldrich catalog number 1603131000), and the optical density at 450 nm (OD_450_) was measured with a ClarioStar plate reader (BMG Labtech, Cary, NC). Endpoint ELISA titers were defined as the first dilution before the OD_450_ dropped below the average signal from PBS-immunized serum at a 1:1,000 dilution.

### Immunofluorescence assay.

iSLK cells were analyzed 2 days postreactivation with 1 to 5 µg/mL doxycycline and 1 mM sodium butyrate, and 293T cells were analyzed 1 to 2 days posttransfection with the plasmid of interest by using BioT transfection reagent (Bioland Scientific LLC, Paramount, CA; catalog number B01). Cells were fixed with 4% paraformaldehyde (Electron Microscopy Sciences; catalog number 15710) in DPBS for 15 min at room temperature without shaking. Cells were washed 3 times with PBS for 5 min and blocked with IFA buffer consisting of DPBS with 10% (vol/vol) heat-inactivated FBS and 3% (wt/vol) BSA for 1 h at room temperature with orbital shaking. For intracellular IFA, IFA buffer contained 0.3% (vol/vol) Triton X-100. Cells were probed with primary antibody diluted in IFA buffer overnight at 4°C on a rocker. After primary incubation, cells were washed 3 times with PBS for 5 min before the addition of 1:2,000 goat anti-mouse IgG(H+L)-Alexa Fluor 594 secondary antibody (Thermo Fisher Scientific; catalog number A11032) diluted in IFA buffer. Cells were covered to protect them from light and incubated with secondary antibody for 2 h at room temperature on an orbital shaker. Secondary antibody was removed, and nuclei were stained with 1:10,000 aqueous Hoescht 33342 solution (Thermo Fisher Scientific; catalog number H3570) diluted in IFA buffer for 10 min at room temperature, protected from light, with orbital shaking. Cells were washed three times for 5 min with PBS before being visualized by fluorescence microscopy.

### Serum antibody removal by cell adsorption.

293T cells were seeded in 10-cm tissue culture dishes (VWR catalog number 10062-880) at a density of 4 × 10^6^ cells. One day after seeding, cells were transfected using BioT with 10 μg of plasmid for the glycoprotein of interest per dish. One day after transfection, cells were washed gently with DPBS, pushed into suspension with DPBS, and split into 3 tubes. Cells were pelleted and kept at 4°C for no longer than 1 to 2 days before use. Serum of interest was diluted 1:2 with DPBS, and 50 μL of the diluted serum was used to resuspend the cell pellet. The mixture was inverted at room temperature for 4 h before the cells were removed by centrifugation at 500 × *g* for 5 min. This was repeated twice for a total of 3 adsorptions per serum sample per cell line. The removal of glycoprotein-specific antibodies was verified by immunofluorescence and ELISA.

### Complement deposition assay.

Lytic replication of KSHV in BC-3-G cells was induced with 20 ng/mL TPA in complete RPMI for 2 days. A total of 100,000 cells were placed in a 96-well V-bottom plate and washed twice with FACS buffer. Cells were then incubated in 50 µL PBS containing experimental serum diluted 1:50 and 10% normal human serum for 30 min at 37°C. Cells were washed twice with FACS buffer and then stained with the following antibodies at a 1:100 dilution in PBS for at least 30 min at 4°C: C1q-PE clone 1A4 (Santa Cruz Biotechnology catalog number sc-53544 PE) and C3b-APC clone 3E7 (Biolegend, San Diego, CA; catalog number 846106). Cells were washed twice and then analyzed on an Attune NxT flow cytometer equipped with a plate reader with single color controls for compensation.

### Statistical analysis.

Statistical analysis was performed with the tests described in the figure legends, using Prism (GraphPad Software, San Diego, CA).

### Data availability.

The mass spectrometry proteomics data have been deposited to the ProteomeXchange Consortium via the PRIDE partner repository (84) with the data set identifier PXD035478. All other reagents used in the manuscript are available from the corresponding author upon reasonable request.
