# Supplementary material for: Immunization of Mice with Virus-Like Vesicles of Kaposi Sarcoma-Associated Herpesvirus Reveals a Role for Antibodies Targeting ORF4 in Activating Complement-Mediated Neutralization
Source: J Virol. 2023 Feb 9;97(2):e01600-22. doi: 10.1128/jvi.01600-22 (PMC9972917; doi:10.1128/jvi.01600-22)
Supplement: Supplemental file 1 — Fig. S1 to S6 and Tables S1 and S2. Download jvi.01600-22-s0001.pdf, PDF file, 1.0 MB [file jvi.01600-22-s0001.pdf]

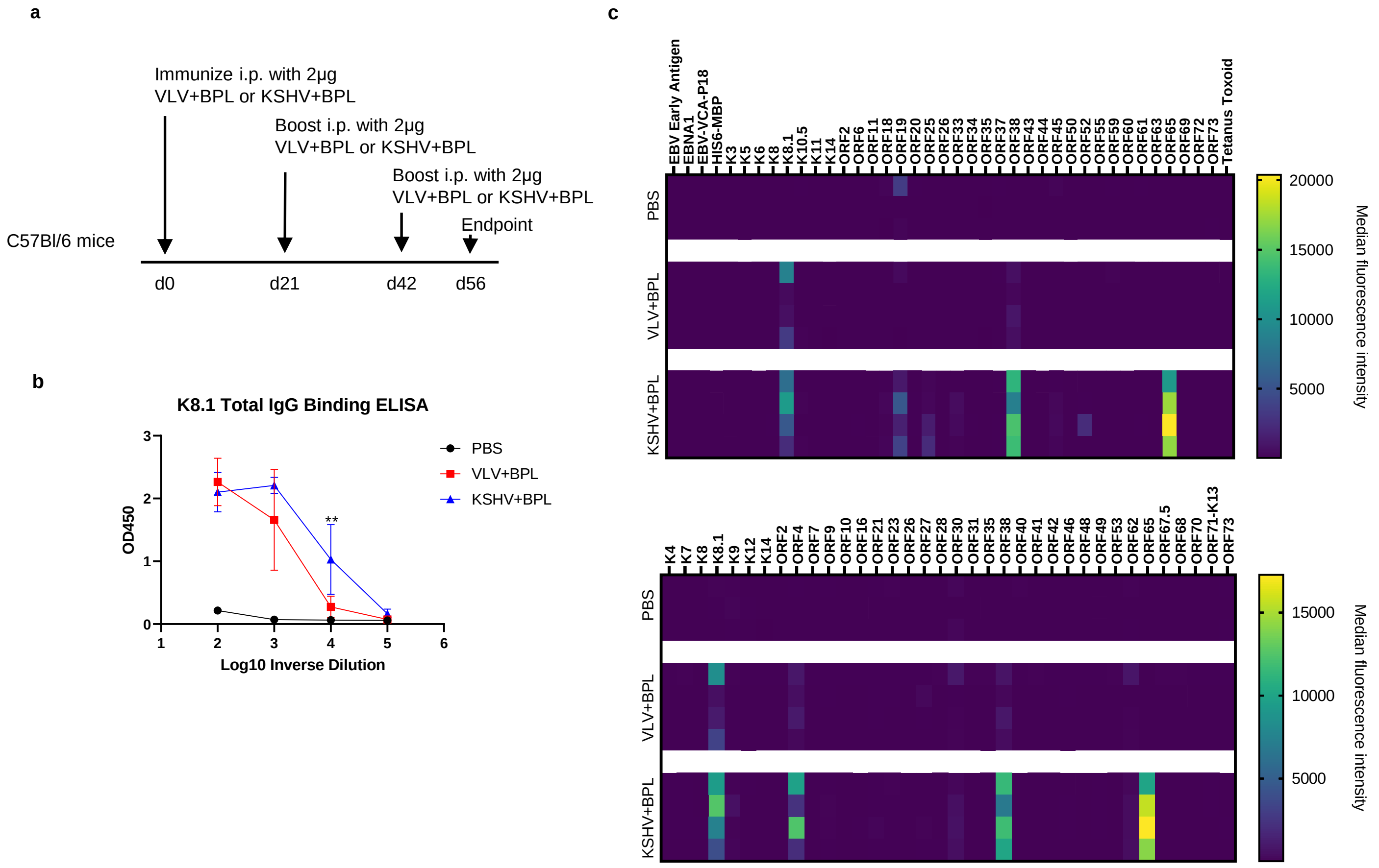

Figure S1: Antibody responses after intraperitoneal immunization of VLVs
(a) Immunization scheme for (b) and (c)
(b) K8.1 ELISA signals from mice immunized with VLV+BPL or KSHV+BPL
(c) Heatmaps showing antibody binding by VLV+BPL or KSHV+BPL immune serum in a bead-based multiplex KSHV antigen assay. Each heatmap represents a different set of beads with some overlapping antigens. Each row represents a different mouse.

Statistical analysis: (b) Two-way ANOVA with Tukey's multiple comparisons. Difference between VLV+BPL and KSHV+BPL is shown. **$p<0.01$. Mean and standard deviation are shown. N=3-4 mice per group

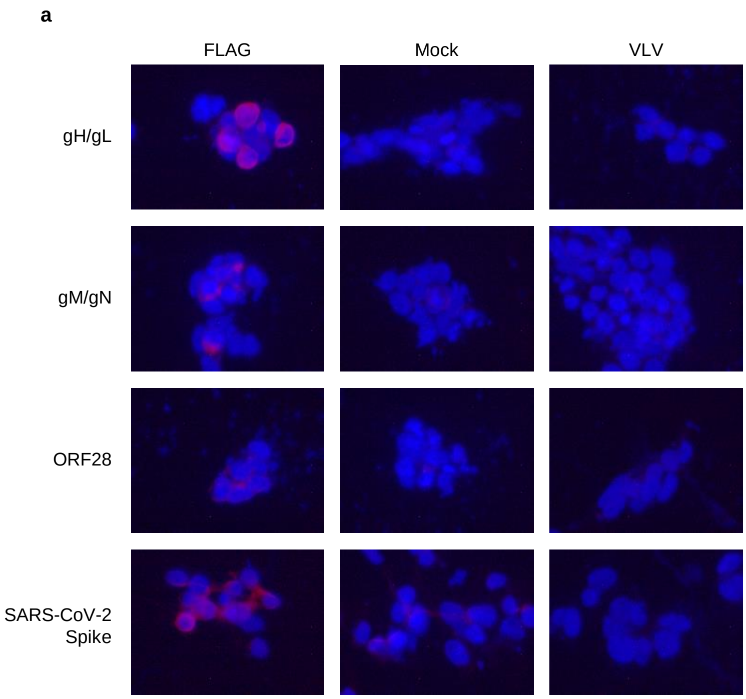

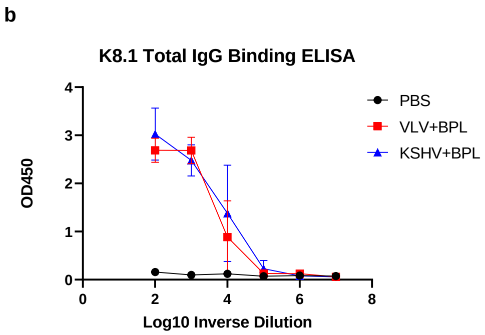

Figure S2: Antibody responses after intramuscular immunization of adjuvanted VLVs
(a-b) Mice were immunized as shown in Figure 2d
(a) Immunofluorescence images of 293T cells expressing KSHV glycoproteins or SARS-CoV-2 Spike stained with pooled mock or VLV+BPL + polyUs-LNP immune serum at a 1:100 dilution
(b) K8.1 ELISA signals from mice immunized with VLV+BPL and KSHV+BPL. Mean and standard deviation are shown. N=3-4 mice per group

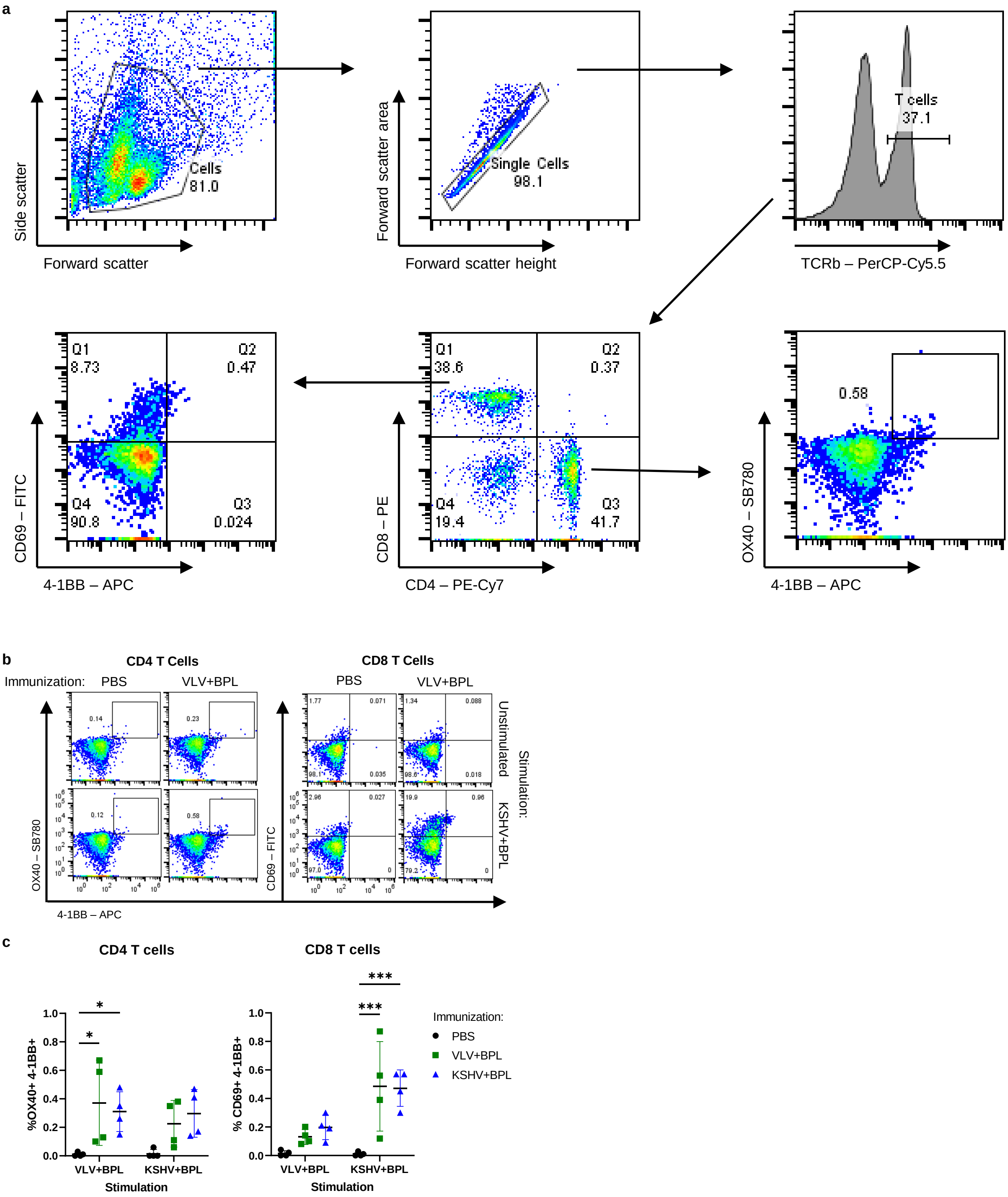

Figure S3: AIM analysis of splenocytes from VLV-immunized mice

(a) Splenocytes were gated for single T cells, separated by CD4 and CD8 expression, and analyzed for AIM markers OX40 and 4-1BB (CD4 cells) or CD69 and 4-1BB (CD8 cells). Mice were immunized as shown in Figure 2d.

(b) Representative flow cytometry plots of CD4 and CD8 T cells used in an AIM assay

(c) Percentages of AIM-positive CD4 and CD8 cells after subtracting background from unstimulated cells

Statistical analysis: (c) Two-way ANOVA with Tukey's test for multiple comparisons. *p<0.05, ***p<0.001. Mean and standard deviation are shown. N=4 mice per group

APC – allophycocyanin; FITC – fluorescein isothiocyanate; PE – R-phycoerythrin; PE-Cy7 – PE-Cyanine7; PerCP-Cy5.5 – Peridinin chlorophyll protein-Cyanine5.5; SB780 – Super Bright 780; TCRb – T cell receptor beta chain

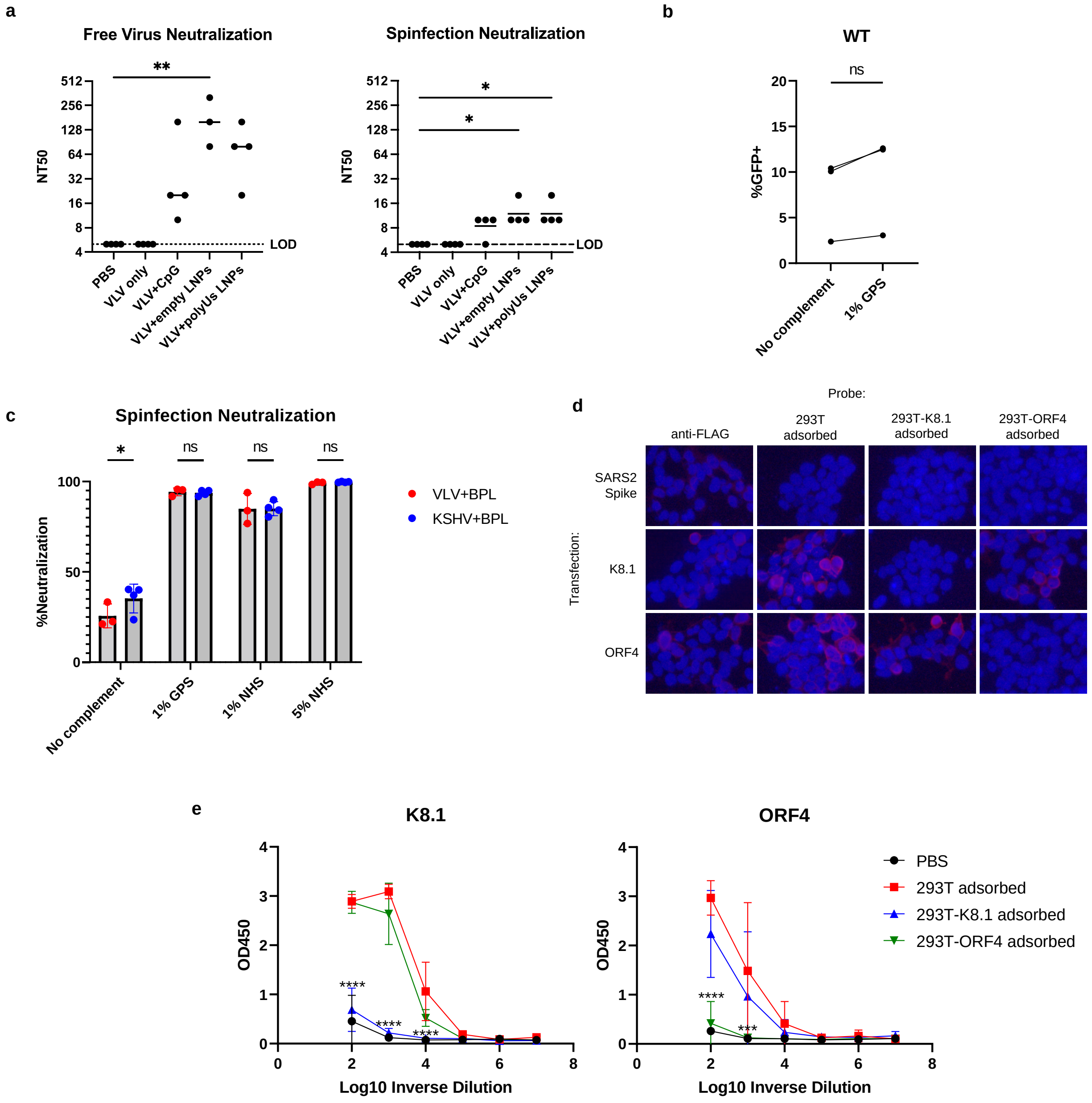

Figure S4: Antibody-dependent complement-mediated neutralization by VLV immune serum

(a) 50% neutralization titers of serum from immunized mice (Fig. 2a). Infection was performed using free virus or spin infection and the titers were calculated as described in Materials and Methods. LOD = limit of detection.

(b) Percentage of GFP+ HEK293 cells infected with WT incubated with heat-inactivated mock (PBS) immune mouse sera at a 1:160 dilution in the presence of complement. Data is pooled from 3 different experiments that used 2 different concentrations of virus.

(c) Heat-inactivated sera from mice immunized as in Fig. 2d were used in complement-mediated spin infection neutralization at 1:80 dilution in the presence of the indicated complement sources.

(d) Representative immunofluorescence images of 293T cells expressing the indicated KSHV glycoprotein and stained with serum at a 1:250 dilution from the VLV+BPL group adsorbed on 293T cells or 293T cells expressing K8.1 or ORF4

(e) K8.1 and ORF4 IgG binding ELISA data from adsorbed sera used in (d)

Statistical analysis: (a) Ordinary one-way ANOVA with Dunnett's test for multiple comparisons against the PBS mock immunized group. Mean is shown. N=3-4 mouse serum samples per group. (b) paired t-test. N=3 different infection experiments. (c) Two-way ANOVA with Tukey's multiple comparisons. Mean and standard deviation are shown. 3 (VLV+BPL) or 4 (KSHV+BPL) mice per group (c). (e) Two-way ANOVA with Tukey's multiple comparisons. Difference between 293T-adsorbed and 293T-K8.1 adsorbed (K8.1 ELISA) or 293T-adsorbed and 293T-ORF4-adsorbed (ORF4 ELISA) are shown. *p<0.05, ** p<0.01. ***p<0.001, ****p<0.0001. Mean and standard deviation are shown. N=3-4 mice per group

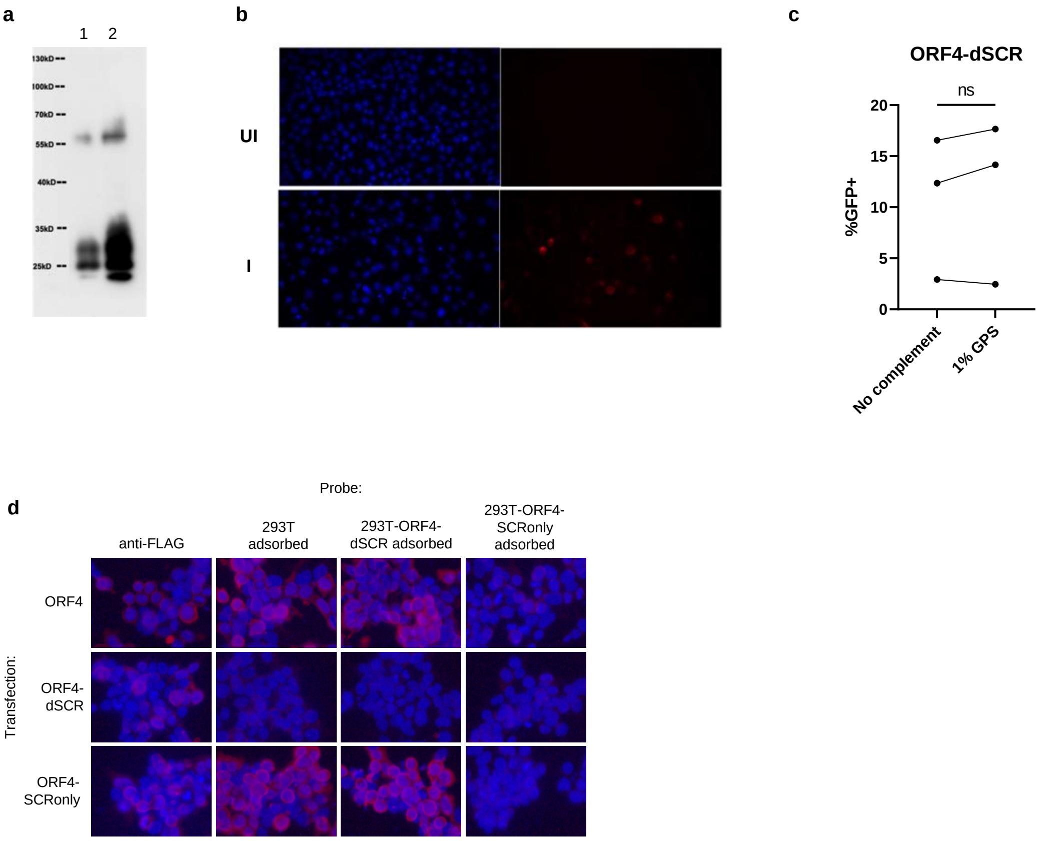

Figure S5: The role of the ORF4 SCR domain on complement-mediated neutralization
(a) iSLK cells infected with ORF4-dSCR KSHV were treated with 1 mM sodium butyrate and 1 µg/mL (lane 1) or 5 µg/mL (lane 2) doxycycline and two days later, total lysates were harvested for western blot analysis using anti-HA antibodies.
(b) IFA analysis of ORF4-dSCR expression upon induction (I) using anti-HA antibodies. Un-induced (UI) cells were included as a control.
(c) Percentage of GFP+ HEK293 cells infected with KSHV ORF4-dSCR incubated with heat-inactivated mock (PBS) immune mouse sera at a 1:160 dilution in the presence of complement. Data is pooled from 3 different experiments that used 2 different concentrations of virus.
(d) Representative immunofluorescence images of 293T cells expressing the indicated KSHV glycoprotein and stained with serum at a 1:250 dilution from the VLV+BPL group adsorbed on 293T cells or 293T cells expressing ORF4-dSCR or ORF4-SCRonly

Statistical analysis: (c) Paired t-test with N=3 different infection experiments.

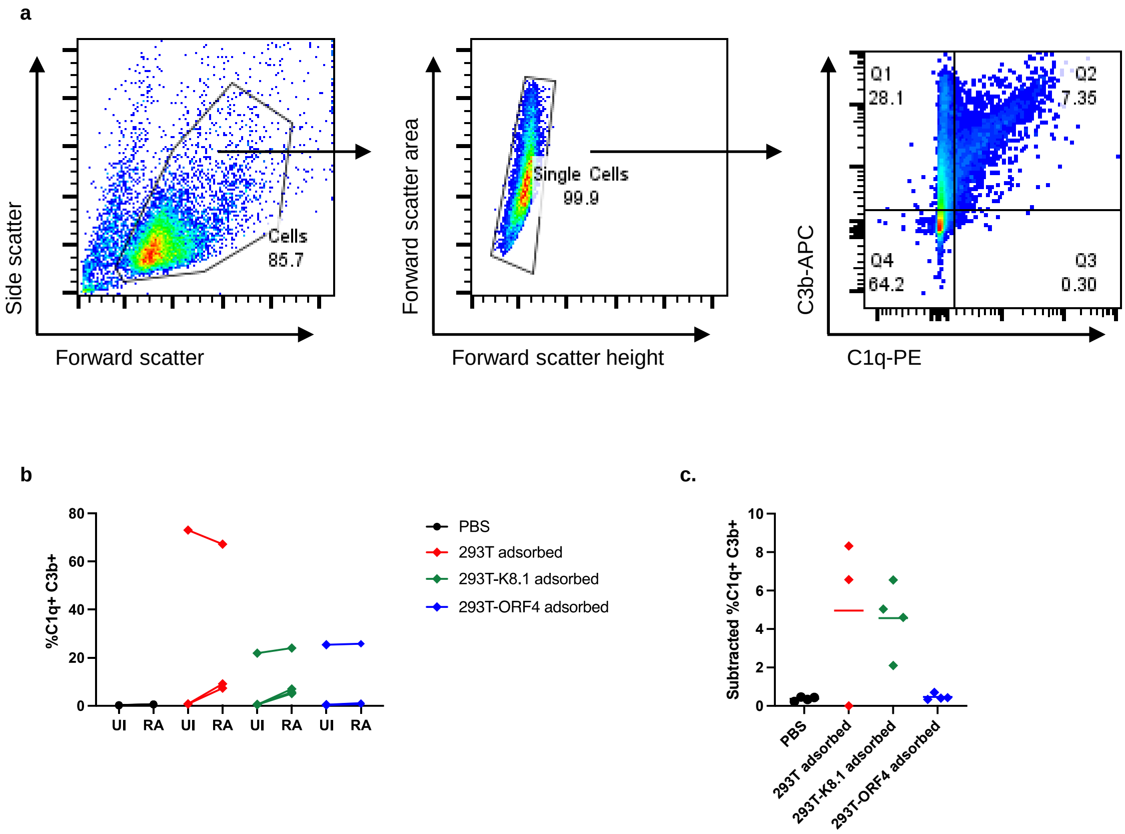

Figure S6: Complement deposition on KSHV-infected B cells

(a) Representative gating strategy for complement deposition assay. BC-3-G cells were gated for live single cells by forward and side scatter and analyzed for C1q and C3b deposition

(b) The VLV immune serum (Fig. 2d) were absorbed with various 293T cells and incubated with BC-3G cells, un-induced (UI) or induced to reactivate latent KSHV (RA) in the present of NHS as a complement source. Percentages of C1q and C3b double positive cells were shown.

(c) Percentages of C1q and C3b double positive reactivated BC-3-G cells after subtracting background from uninduced BC-3-G cells.

Statistical analysis: (b) One way ANOVA with Šídák's multiple comparisons test. No significance was found. (c) One way ANOVA with Tukey's multiple comparisons test. No significance was found. N=3 or 4 mice per group

APC – allophycocyanin; PE – R-phycoerythrin

Protein	VLV/KSHV Intensity Ratio	Function
**Capsid and capsid-associated proteins**		
ORF25	0.051	Major capsid protein
ORF26	0.038	Triplex protein
ORF17.5	0.202	Capsid protein
ORF65	0.051	Small capsid protein
ORF62	0.029	Triplex protein
ORF32	0.088	Binds capsid
**Envelope proteins**		
ORF8	0.647	Glycoprotein B
ORF4	0.603	Complement control
ORF28	0.419	EBV gp150 homolog
ORF22	0.396	Glycoprotein H
ORF39	0.394	Glycoprotein M
ORF47	0.354	Glycoprotein L
K8.1	1.044	Glycoprotein K8.1
**Tegument proteins**		
ORF75	0.311	Tegument protein, inhibition of ND10 innate immunity
ORF52	0.419	Tegument protein, viral egress, inhibition of cGAS
ORF64	0.423	Tegument protein, inhibition of RIG-I
ORF21	0.395	Thymidine kinase
ORF63	0.399	Tegument protein, inhibition of inflammasome
ORF42	0.587	Tegument protein, viral egress
ORF55	0.684	Tegument protein
ORF67	0.539	Tegument protein
**Other detected proteins**		
ORF45	0.423	Viral egress, inhibition of IRF7
ORF33	0.372	Viral egress
ORF38	0.385	Viral egress
ORF27	0.640	Cell to cell spread
ORF11	0.337	EBV LF2 homolog
ORF59	0.733	Viral processivity factor
ORF68	0.703	Genome packaging
ORF23	0.397	RNA polymerase pre-initiation complex
ORF60	0.720	Viral ribonucleotide reductase subunit
ORF61	0.481	Viral ribonucleotide reductase subunit
K5	0.517	MHC-I downregulation
ORF6	0.706	ssDNA binding protein

Table S1: Viral proteins found in VLV and KSHV preparations
The viral proteins found in the top 1200 proteins identified by average intensity in VLV preparations, organized by function.

UniprotKB Accession No.	VLV/KSHV Intensity Ratio	Protein Symbol	Protein Description
P63261	0.644	ACTG	Actin, cytoplasmic 2
P07355	0.683	ANXA2	Annexin A2
Q09666	0.706	AHNK	Neuroblast differentiation-associated protein
P08195	0.697	4F2	4F2 cell-surface antigen heavy chain
P08238	0.644	HS90B	Heat shock protein HSP 90-beta
P68104	0.629	EF1A1	Elongation factor 1-alpha 1
P21333	0.829	FLNA	Filamin-A
P14618	0.563	KPYM	Pyruvate kinase
P04406	0.943	G3P	Glyceraldehyde-3-phosphate dehydrogenase
P11142	0.660	HSP7C	Heat shock cognate 71 kDa protein
P0DMV8	0.666	HSP1A	Heat shock 70 kDa protein 1A
P68363	0.681	TBA1B	Tubulin alpha-1B chain
P23528	0.787	COF1	Cofilin-1
P68371	0.737	TBB4B	Tubulin beta-4B chain
Q06830	0.778	PRDX1	Peroxiredoxin-1
P08133	1.441	ANXA6	Annexin A6
P08670	0.801	VIME	Vimentin
P08758	0.859	ANXA5	Annexin A5
P05023	0.657	AT1A1	Sodium/potassium-transporting ATPase subunit alpha-1
P21980	0.660	TGM2	Protein-glutamine gamma-glutamyltransferase 2
P13639	0.661	EF2	Elongation factor 2
P00338	0.794	LDHA	L-lactate dehydrogenase A chain
P35579	0.713	MYH9	Myosin-9
Q562R1	0.640	ACTBL	Beta-actin-like protein 2
O75369	0.691	FLNB	Filamin-B
P05556	0.542	ITB1	Integrin beta-1
P49327	0.768	FAS	Fatty acid synthase
P02751	0.594	FINC	Fibronectin
P69905	0.774	HBA	Hemoglobin subunit alpha
P62937	0.693	PPIA	Peptidyl-prolyl cis-trans isomerase A

Table S2: Cellular proteins found in VLV and KSHV preparations
The top 30 cellular proteins as determined by average intensity in VLV preparations.
